# Liver-specific amino acid metabolism impacts on efficacy of cancer immunotherapy

**DOI:** 10.1038/s41388-026-04023-0

**Published:** 2026-10-17

**Authors:** Yuma Fukumoto, Fumiaki Mukohara, Momoko Iwatsuru, Youki Ueda, Hiroaki Kanzaki, Sadahisa Ogasawara, Jason Lin, Masahito Kawazu, Toshifumi Ninomiya, Naoya Kemmotsu, Joji Nagasaki, Shinichi Toyooka, Tsuyoshi Osawa, Takamasa Ishino, Yosuke Togashi

**Affiliations:** 1 Department of Tumor Microenvironment, Graduate School of Medicine Dentistry and Pharmaceutical Sciences, Okayama University, Okayama, Japan; 2 Department of General Thoracic Surgery and Breast and Endocrinological Surgery, Dentistry and Pharmaceutical Sciences, Okayama University Graduate School of Medicine, Okayama University, Okayama, Japan; 3 Department of Gastroenterology, Graduate School of Medicine, Chiba University, Chiba, Japan; 4 Division of Cell Therapy, Chiba Cancer Center Research Institute, Chiba, Japan; 5 Department of Neurological Surgery, Okayama University Graduate School of Medicine, Dentistry and Pharmaceutical Sciences, Okayama University, Okayama, Japan; 6 Division of Nutriomics and Oncology, RCAST, The University of Tokyo, Tokyo, Japan; 7 Department of Chemistry and Biotechnology, Graduate School of Engineering, The University of Tokyo, Tokyo, Japan; 8 Department of Biological Sciences, Graduate School of Science, The University of Tokyo, Tokyo, Japan; 9 Department of Respiratory Medicine, Okayama University Hospital, Okayama, Japan; 10 Kindai University Faculty of Medicine, Osaka, Japan

**Keywords:** Liver cancer, Immunosurveillance

## Abstract

The combination immunotherapies, including those with atezolizumab and bevacizumab (Atez/Bev), have become standard therapy for hepatocellular carcinoma (HCC). However, its efficacy remains limited and reliable biomarkers are lacking. In this study, we analyzed paired tumor and background liver tissues from Atez/Bev-treated HCC patients and found that high programmed cell death-1 (PD-1) expression in CD8^+^ tumor-infiltrating lymphocytes (TILs) correlated with therapeutic response independent of etiology. RNA sequencing on the tumor samples revealed that the branched-chain amino acid (BCAA)-related metabolic pathway was enriched in the group with high PD-1 expression in CD8⁺ TILs. Along with in vitro experiments, we identified the importance of BCAAs for activation and differentiation of CD8^+^ T cells. In addition, BCAA metabolism was related to response to PD-1 blockade in not subcutaneous but intrahepatic mouse model–specific manner. Actually, public dataset analyses revealed that high expression of *BCAT1*, a key enzyme in BCAA metabolism, was associated with poor prognosis in HCC, but not in other cancer types. These findings suggest the importance of liver-specific BCAA metabolism in antitumor immunity and highlight the need to assess the tumor microenvironment within its organ-specific metabolic context.

**Figure:**
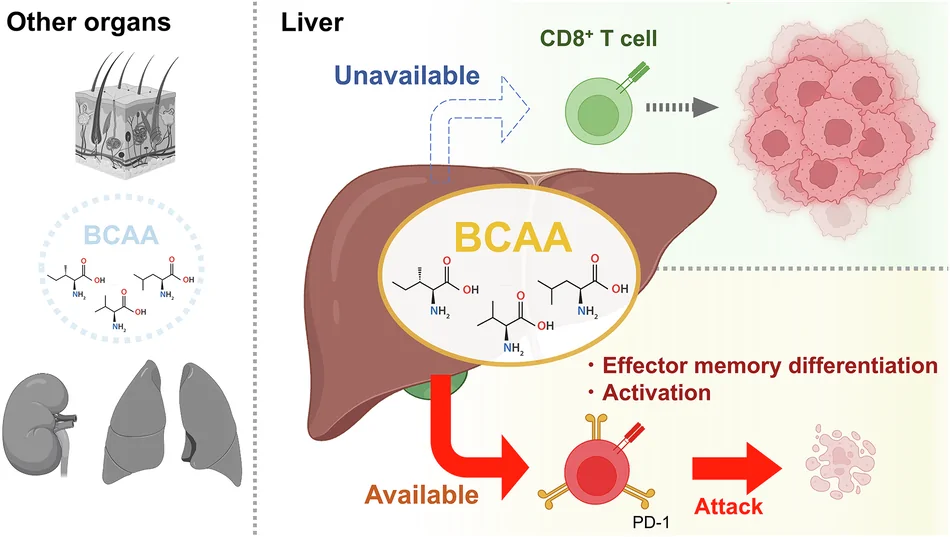

## Plain language summary

While cancer immunotherapy has advanced liver cancer treatment, many patients still do not experience long-term benefits. In this study, we analyzed samples from liver cancer patients and found that branched-chain amino acids (BCAAs), key nutrients found in the liver, are essential for cancer-fighting immune cells to do their job. We demonstrated that these immune cells and cancer cells actively compete for BCAAs within the liver. This competition can weaken the immune cells, making the treatment less effective. Our findings highlight that to improve cancer therapies, we need to understand how the unique metabolic environment of a specific organ affects the body’s immune response.

## Introduction

Liver cancer, over 90% of which are hepatocellular carcinoma (HCC), is the sixth most common cancer and the fourth leading cause of cancer-related death globally [1]. HCC generally occurs from chronic hepatitis or cirrhosis caused by various risk factors including infection of hepatitis B/C viruses (HBV/HCV), alcohol consumption, and metabolic dysfunction associated steatotic liver disease (MASLD) [1]. Many HCC patients are initially diagnosed at advanced stages, and early-stage cases often advance due to high recurrence rates, emphasizing the urgent need for effective systemic therapies [1]. However, systemic therapy for HCC was long limited to vascular endothelial growth factor (VEGF)-targeted tyrosine kinase inhibitors, such as sorafenib which provided a progression-free survival (PFS) of less than 6 months [2]. Although the combination of atezolizumab and bevacizumab (Atez/Bev) recently demonstrated superior antitumor activity to sorafenib and became a standard therapy [3], its efficacy remains suboptimal with a response rate of less than 50% [3]. Therefore, identification of predictive biomarkers and development of therapies to overcome resistance are warranted.

Immune checkpoint inhibitors (ICIs) reinforce antitumor immunity by blocking suppressive immune checkpoints such as programmed cell death-1 (PD-1), programmed cell death-ligand 1 (PD-L1), and cytotoxic T lymphocyte-associated protein 4 (CTLA-4) [4, 5]. Among various immune cell subsets, CD8^+^ T cells are particularly critical in antitumor immunity within the tumor microenvironment (TME) and key targets of ICI therapy [4–6]. Though T cells require metabolic substrates such as glucose and amino acids (AAs) for activation and differentiation [7], cancer cells also consume large amounts of these substrates to support their rapid proliferation [8]. Consequently, it is known that metabolic competition for these substrates occurs between T cells and cancer cells, and T cells cannot activate sufficiently within a nutrient-deprived TME [8–11]. While liver is responsible for metabolism [12], most reports on metabolic TME remain limited to experimental investigations in HCC [9–11, 13].

In this study, we analyzed immune status of both tumor and background liver tissues from patients with HCC who received Atez/Bev therapy. Along with RNA sequencing (RNA-seq), we found the importance of branched chain amino acids (BCAAs) in the activation of tumor-infiltrating lymphocytes (TILs) from in vitro experiments. In addition, in vivo experiments revealed liver-specific metabolic competition for BCAAs between cancer cells and T cells in the TME, which can be related to ICI efficacy. These findings implicate liver-specific BCAA metabolism as potential biomarkers and therapeutic targets for liver tumors. Furthermore, we highlight the importance of evaluating the TME in the context of organ-specific metabolism, which can lead to identifying better biomarkers and developing novel therapies based on metabolic perspectives.

## Methods

### Patients and samples

Fifty patients with advanced HCC who underwent RNA-seq and flow cytometry (FCM) analysis on samples biopsied prior to Atez/Bev treatment at Chiba University Hospital between 2020 and 2022 were enrolled in this study (Table S1) [14]. Of the 50 patients, background liver tissues from 47 patients and peripheral blood from 44 patients were available for analysis due to the sample limitation. Clinical data from patients in this study was obtained retrospectively and the data were locked on March 31, 2023.

### Clinical sample preparation for FCM

For clinical sample preparation for FCM, biopsy samples were enzymatically digested with Tumor & Tissue Dissociation Reagent (TTDR) (BD Biosciences, Franklin Lakes, NJ, Cat# 661563). Peripheral blood mononuclear cells (PBMCs) were isolated by density gradient centrifugation with Lymphocyte Separation Solution (NACALAI TESQUE, INC., Kyoto, Japan, Cat# 20828‑44).

### FCM

FCM was performed as previously described [15]. Briefly, cells were washed with phosphate-buffered saline (FUJIFILM Wako Pure Chemical Corporation, Cat# 048-29805) containing 2% fetal bovine serum (FBS) (Cytiva, Tokyo, Japan, Cat# SH30070.03) and subjected to staining with surface antibodies. Intracellular staining was performed with specific antibodies and the Fixation/Permeabilization Buffer Set (Thermo Fisher Scientific, Waltham, MA, Cat# 88-8824-00), according to the manufacturer’s instructions. For analysis of phosphorylated proteins, cells were fixed using intracellular fixation buffer (Thermo Fisher Scientific, Cat# 00-8222-49), permeabilized with methanol according to the manufacturer’s instructions and stained with specific antibodies. For murine intracellular cytokine staining, GolgiPLUG reagent (BD Biosciences, Cat# 555029) was added for the last 6 h of culture with anti-CD3 monoclonal antibody (mAb) (5 μg/mL 145-2C11, Biolegend, Cat# 100359) and anti-CD28 mAb (2 μg/mL 37.51, Biolegend, Cat# 102116). The samples were assessed using a BD FACS Fortessa instrument (BD Biosciences, RRID: SCR_001456) and FlowJo software (BD Biosciences, RRID: SCR_008520). The stained antibodies were diluted according to the manufacturer’s instructions. Detailed information on the antibodies used is presented in Table S2.

### RNA-seq

Total RNA samples were extracted from fresh frozen tissue using AllPrep DNA/RNA Mini Kit (Qiagen, Hulsterweg, Netherlands, Cat# 80204) or from three to five 10-μm-thick formalin fixed paraffin embedded (FFPE) tissue sections by RNeasy FFPE Kit (Qiagen, Hilden, Germany, Cat# 73504). Then, they were subjected to RNA-seq library preparation with SMART-Seq Stranded Kit (Takara Bio Inc., Shiga, Japan. Cat# 634442) and sequenced with NovaSeq 6000 (Illumina, San Diego, CA, RRID: SCR_016387) (150 bp paired-end) according to the manufacturer’s instructions. The resultant paired raw sequencing reads were processed and analyzed as previously reported [14].

### Gene set enrichment analysis (GSEA)

Enriched pathways were determined using our RNA-seq for tumor samples based on the GSEA tool available from the Broad Institute website (http://software.broadinstitute.org/gsea/index.jsp, RRID: SCR_003199) [16].

### Publicly-available dataset analysis

RNA-seq data of 181 HCC patients treated with Atez/Bev therapy in the IMbrave150 study were obtained from the European Genome-phenome Archive (https://ega-archive.org/) under accession number EGAD00001008128 and EGAD00001008130 [17, 18]. In this cohort, the responders were defined as patients with PFS of 6 months or longer. Adapter sequences were trimmed from the raw RNA-seq reads using fastp software (v0.23.4, RRID: SCR_016962). The processed reads were aligned to the human reference genome (GRCh38) using STAR software (v2.7.11b, RRID: SCR_004463). Gene-level expression quantification was performed with RSEM software (v1.3.1, RRID: SCR_000262). Data visualization was performed using the enrichplot packages (v1.26.1, RRID: SCR_026996) in R software.

RNA-seq data and survival data for hepatocellular carcinoma (*n* = 373), melanoma (*n* = 462), lung squamous carcinoma (*n* = 486), lung adenocarcinoma (*n* = 508) and kidney cancer (*n* = 496) were acquired from The Cancer Genome Atlas (TCGA) database (Firehose Legacy version) published in cBioPortal (https://www.cbioportal.org/, RRID: SCR_014555) [19, 20]. For each cancer type, patients were stratified into high and low *BCAT1* expression groups based on the median *BCAT1* mRNA expression level. Overall survival (OS), the period from the date of diagnosis until the date of death from any cause, was analyzed using Kaplan–Meier curves and compared with the log-rank test.

### Quantification of BCAA concentration

BCAA concentrations of murine organs, medium, or tumors were determined using the Branched Chain Amino Acid Assay kit (Cell Biolabs Inc., San Diego, CA, Cat# MET-5056) following the manufacturer’s instructions. BCAA concentrations were measured in triple technical replicates against negative control samples lacking leucine dehydrogenase and calculated based on the leucine standards.

To evaluate the consumption of BCAA by cells, 2 × 10^5^ cells were cultured for 48 h and the BCAA levels in DMEM medium (Fujifilm Wako Pure Chemical Corporation, Cat# 044‑29765) supplemented with 10% FBS were measured. BCAA consumption was assessed by calculating the difference in BCAA concentrations between the culture medium before and after incubation.

### In vitro human T cell chronic stimulation model

3 × 10^5^ naive CD8^+^ T cells were isolated from PBMCs of healthy donors using the MojoSort™ Human CD8 Naive T Cell Isolation Kit (BioLegend, SanDiego, Cat# 480045). They were cultured in complete RPMI 1640 medium, 20 individual AA-deprived medium, or all AA-deprived medium supplemented with 10% human AB serum (MP Biomedicals, Irvine, CA, Cat# 092930949), antibiotics, and recombinant human interleukin-2 (300 IU/mL, PeproTech, Cranbury, NJ, Cat# 200-02). They were stimulated with anti-CD3 monoclonal antibody (mAb) (2.5 μg/mL OKT-3, BD Biosciences, RRID: AB_2869821) and anti-CD28 mAb (2.5 μg/mL CD28.2, Thermo Fisher Scientific, RRID: AB_468926) every 48 h for 3 times to mimic chronic antigen stimulation and subjected to the analyses [21]. For analysis of phosphorylated proteins, cells were stimulated with anti-CD3/CD28 mAbs for 30 min before staining for the FCM analysis. To stimulate the upstream pathway of mammalian target of rapamycin complex 1 (mTORC1) signaling, recombinant human insulin-like growth factor I (rhIGF-I, 100 ng/mL, Biolegend, Cat# 588604) was added from the third round of anti-CD3/CD28 stimulation, followed by subsequent downstream analyses.

### Measurement of ATP derived from mitochondrial metabolism and glycolysis

Total and glycolytic ATP were measured with ATP Assay Kit-Luminescence (Dojindo, Kumamoto, Japan, Cat# 346-09793). The luciferase activity was measured using Flex Station 3 (Molecular Devices, Sunnyvale, CA, Cat# 0200-6182) at the Central Research Laboratory, Okayama University Medical School. To analyze glycolytic ATP, cells were incubated with 2.5 μM oligomycin A (Adipogen Life Sciences, Fuellinsdorf, Switzerland. Cat# AG‑CN2‑0517) for 3 h prior to the measurement. Mitochondrial ATP was calculated as the difference between total and glycolytic ATP.

### Cell lines

The Hepa1-6 (murine HCC, RRID: CVCL_0327) cell line was obtained from the RIKEN BRC (Tsukuba, Japan). The MC-38 (murine colon cancer, RRID: CVCL_B288) cell line was purchased from Kerafast (Boston, MA) and EMT6 (murine breast cancer, RRID: CVCL1923) cell line was purchased from American Type Culture Collection (Manassas, VA). These cell lines were recently obtained and maintained in DMEM medium supplemented with 10% FBS. All cells were confirmed to be free of *Mycoplasma* using a polymerase chain reaction (PCR) Mycoplasma Detection Kit (TaKaRa Bio Inc. Cat# 6601), according to the manufacturer’s instructions.

### Constructs and transfection

The retroviral vector carrying mock (Cat# VB200123-1020exq), murine *Bcat1* (Cat# VB230903-1225qem) and *Bcat2* (Cat# VB260126-1224xjb) were purchased from VectorBuilder (Chicago, IL). Each of these vectors and the pVSV-G vector (TaKaRa Bio Inc. Cat# S1958) were transfected into packaging cells using Lipofectamine 3000 (Thermo Fisher Scientific, Cat# L3000075). After 48 h, the supernatant was concentrated and transduced into cells.

The lentiviral vectors carrying non-targeting (Cat# VB010000-9460fht) and murine *Bcat1*-targeting short hairpin RNA (shRNA) (Cat# VB230824-1113yzm) were purchased from VectorBuilder. Lentiviral vector pHAGE PGK-GFP-IRES-LUC-w (Addgene plasmid #46793; http://n2t.net/addgene:46793; RRID: Addgene_46793) carrying luciferase was gifted from Darrell Kotton (Addgene, Watertown, MA) [22]. Each vector was transfected into packaging cells using Lipofectamine 3000 Reagent with pMDLg/pRRE (Addgene plasmid #12251; http://n2t.net/addgene:12251; RRID: Addgene_12251), pRSV-Rev (Addgene plasmid #12253; http://n2t.net/addgene:12253; RRID: Addgene_12253), and pMD2.G (Addgene plasmid #12259; http://n2t.net/addgene:12259; RRID: Addgene_12259), which were gifted by Didier Trono (Addgenee, Watertown, MA) [23]. After 48 h, the supernatant was concentrated and transduced into the cells.

### Reverse transcription quantitative PCR (RT-qPCR)

Total RNA was isolated with NucleoSpin RNA Plus (TaKaRa Bio Inc. Cat# 740984.10) and was reverse transcribed into cDNA using PrimeScript RT Master Mix (TaKaRa Bio Inc. Cat# RR036A), and RT-qPCR was performed using TB Green Premix Ex Taq II (TaKaRa Bio Inc. Cat# RR820S) according to the manufacturer’s instructions. Murine *Actb* was used as an internal control. Real-time PCR was conducted using StepOnePlus (Applied Biosystems, Foster City, CA, RRID: SCR_015805) at the Central Research Laboratory, Okayama University Medical School. The experiments were performed in triplicate. The primers used are listed in Table S3.

### Cell proliferation assay

Cells were cultured in a 96-well plate. After culturing for 48 h, the number of cells was evaluated by using Cell counting Kit-8 (Dojindo Laboratories, Cat# 343-07623) according to the manufacturer’s instructions. The 450 nm absorbance was measured using a Multiskan FC microplate photometer (Thermo Fisher Scientific, Cat# 51119000).

### Mouse strains

Female C57BL/6J mice (6–8 weeks old) were purchased from SLC Japan (Shizuoka, Japan, RRID: MGI:5488963) for the syngeneic mouse model using Hepa1-6 and MC-38 cells. For the syngeneic mouse model using EMT6 cells, female BALB/c mice (6–8 weeks old) were purchased from SLC Japan (RRID: MGI:2161019).

### In vitro mouse coculture assay

2 × 10^5^ Hepa1-6 cells were cocultured with 2 × 10^5^ CD8^+^ T cells isolated from spleens of female C57BL/6J mice (6–8 weeks old) using the MojoSort™ Mouse CD8 T Cell Isolation Kit (Biolegend, San Diego, Cat# 480035). The coculture assays were performed for 48 h in the presence of 0.5 μg/mL anti-CD3 and 10 μg/mL anti-CD28 mAbs, and 30 IU/mL IL-2 (PeproTech), with or without BCAAs (L-Isoleucine (Sigma-Aldrich, St. Louis, MO, Cat# I7403) 0.38 mM, L-Leucine (Sigma-Aldrich, Cat# L8912) 0.38 mM, L-Valine (Sigma-Aldrich, Cat# V0513) 0.17 mM), and subjected to the analyses.

### In vivo subcutaneous model

1 × 10^6^ cells were injected subcutaneously (day 0), and the tumor volume was monitored twice a week. The means of the long and short tumor diameters were used to generate tumor growth curves. When the tumor volume reached approximately 100 mm³ on day 5, mice were randomly assigned using a simple randomization method to receive either anti-PD-L1 mAb (clone 10 F.9G2; Selleck Chemicals, Houston, TX; Cat# A2115) (200 μg/mouse) or control mAb (clone RTK2758; BioLegend; RRID: AB_326523). Each antibody was intraperitoneally (i.p.) administered three times every 3 days. Tumors were harvested 7 days after treatment initiation for TIL analysis. The in vivo experiments were performed at least twice.

### In vivo intrahepatic model

1 × 10^6^ cells were injected into the left lobe of liver (day 0). On day 5, the mice were randomly assigned using a simple randomization method to receive either anti-PD-L1 mAb or control mAb, which was i.p. administered three times every 3 days. On day 12, luciferase-transduced MC-38 tumors bearing mice were i.p. administered 150 mg/kg D-Luciferin (Summit Pharmaceuticals International, Tokyo, Japan, Cat# XLF-1) under anesthesia. Bioluminescent images were obtained using IVIS Imaging System (Xenogen, Alameda, CA, USA). Radiance was averaged within the region of interest at the plateau state using Living Image 3.0 (Xenogen, RRID: SCR_014247). The excised tumors were digested with the TTDR reagent for TIL analysis. To feed BCAAs, mice were provided with drinking water containing valine (15 mg/mL), leucine (15 mg/mL), and isoleucine (15 mg/mL) throughout the entire experimental period, whereas normal-BCAA groups received sterile distilled water, as described previously [24]. The in vivo experiments were performed at least twice.

### Statistical analyses

GraphPad Prism 9 (GraphPad Software, San Diego, CA, RRID: SCR_002798) was used for statistical analyses. In vitro experiments were conducted in triplicate, and in vivo experiments were performed at least twice using five mice per group, with investigators blinded to group allocation. Data distribution and variance were not formally tested because of the small sample sizes. PFS was defined as the duration from the initiation of Atez/Bev combination therapy to the first documented disease progression or death from any cause and OS was defined as the duration from the initiation of Atez/Bev combination therapy to death from any cause. PFS and OS were investigated using the Kaplan–Meier method, with group comparisons performed via the log-rank test. The relationships of continuous variables between two groups and among multiple groups were analyzed using unpaired two-tailed Student’s *t*-test and one-way analysis of variance (ANOVA), respectively. To evaluate the predictive value for responders, receiver operating characteristic curves were generated for continuous variables by plotting the true-positive rate against the false-positive rate across various threshold settings. Area under the curve presented in each plot summarizes the predictive performance of the continuous variables. Cutoff values for the continuous variables were determined as the thresholds that maximized the sum of sensitivity and specificity. The correlation between two variables was evaluated by computing Pearson’s correlation coefficient. The relationships among tumor volume curves were compared using two-way ANOVA. For multiple comparisons testing, Bonferroni corrections were used. All tests were two-tailed, and statistical significance was set at *P* < 0.05. All statistical details are provided in the figure legends.

## Results

### High PD-1 expression in CD8^+^ T cells in the hepatic TME is important for efficacy of cancer immunotherapy

Among a total of 94 patients with HCC who received Atez/Bev combination therapy between October 2020 and December 2022 at Chiba University Hospital, we evaluated pre-treatment clinical samples from 50 patients using both FCM and RNA-seq [14]. The patient characteristics of the 50 cases are summarized in Table S1. All patients had good performance status (0 or 1) with a median age of 74 years, and 39 patients (78%) were male. Of the patients, 10 (20%) had HBV-related HCC, 15 (29%) had HCV-related HCC, and 25 (50%) had non-B non-C (NBNC)-HCC. At baseline, macrovascular invasion and extrahepatic metastasis were each identified in 17 patients (34%). Many patients (*n* = 42, 84%) received Atez/Bev as first-line therapy (Table S1). The median OS and PFS were 691 and 255 days, respectively, which are similar to a previous study [3]. Though NBNC-HCC was previously reported to have a worse prognosis by cancer immunotherapy [13], no significant differences in both OS and PFS were observed among HBV, HCV, and NBNC hepatitis in our cohort (Fig. S1A, B).

**Fig. 1:**
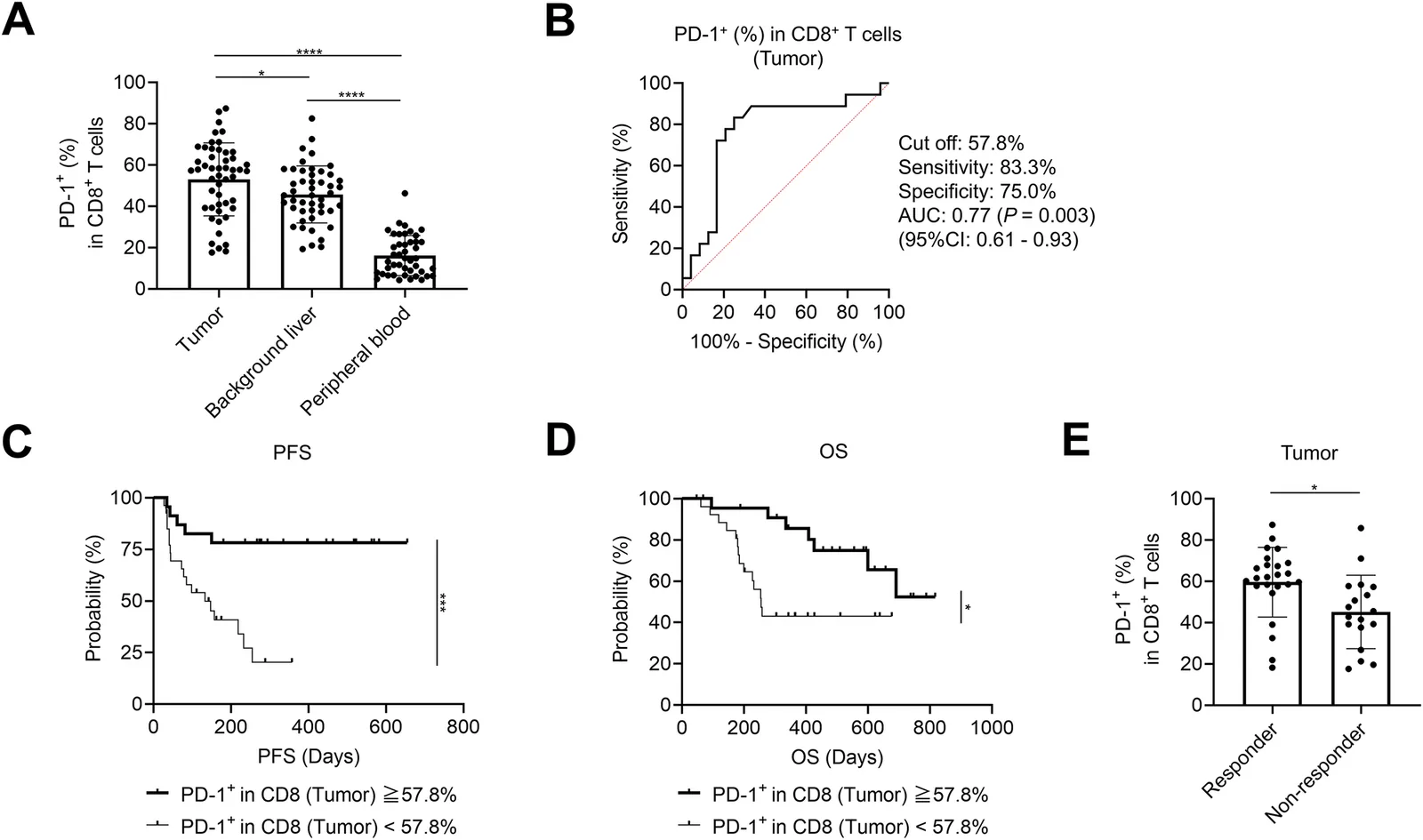
Flow cytometry (FCM) data and clinical outcomes in patients with hepatocellular carcinoma (HCC) treated with atezolizumab and bevacizumab (Atez/Bev). **A** PD-1 expression in CD8^+^ T cells of tumor tissues, background liver tissues and peripheral blood. Biopsy samples underwent brief enzymatic digestion, and peripheral blood mononuclear cells were isolated by density gradient centrifugation. Lymphocytes were stained and analyzed by FCM. Summary is shown. **B** Receiver operating characteristic (ROC) curve for PD-1^+^ (%) in CD8^+^ T cells of the tumor tissues to predict responders. Progression-free survival (PFS) was defined as the duration from the initiation of therapy to the first documented disease progression or death from any cause, and patients whose PFS was 6 months or longer were defined as responders. Kaplan–Meier curves for PFS (**C**) and overall survival (OS) (**D**) stratified by PD-1 expression in CD8^+^ T cells of tumor tissues. OS was defined as the time from initiation of therapy to death from any cause. To compare prognosis, the cutoff of 57.8% for PD-1^+^ (%) in CD8^+^ T cells, determined by the ROC analysis in (**B**), was used. **E** PD-1 expression in CD8^+^ T cells of tumor tissues according to response. The analyses were performed as described in (**A**) and responders were defined as described in (**B**). The summary is shown. One-way analysis of variance with the Bonferroni correction was used in (**A**), the log-rank test was used to compare Kaplan–Meier curves in (**C**, **D**), and unpaired two-tailed Student’s *t*-test was used in (**E**) for statistical analyses. AUC area under the curve; ^*^P < 0.05; ^**^P < 0.01; ^****^P < 0.0001.

While previous reports have shown that CD8^+^ T cells play a crucial role and that PD-1 expression in CD8^+^ TILs can predict ICI efficacy [4–6, 25, 26], another report has shown that PD-1^+^CD8^+^ T cells in the background liver of NBNC hepatitis contribute to poor prognosis in patients with HCC treated with ICIs [13]. Therefore, we evaluated PD-1^+^CD8^+^ T cells in the background liver tissues in addition to tumor tissues using FCM. Consistent with a previous study [27], PD-1 expression in CD8^+^ T cells was the highest in the TME followed by the background liver tissues and peripheral blood (Fig. 1A). No correlation was observed in PD-1 expression of CD8^+^ T cells among tumor, background liver and peripheral blood (Fig. S2A–C). In addition, we could predict responders to Atez/Bev therapy with PFS of 6 months or longer, using PD-1 expression in CD8^+^ TILs, as previously reported (Fig. 1B) [25]. Indeed, patients with high PD-1 expression in CD8^+^ TILs had significantly longer PFS and OS than those with low PD-1 expression (Fig. 1C, D) and PD-1 expression in CD8^+^ TILs was higher in responders (Fig. 1E), whereas PD-1 expression in CD8^+^ TILs was comparable across different etiologies (Fig. S2D). Conversely, PD-1 expression in CD8^+^ T cells from the background liver tissues was not predictive of treatment efficacy and was comparable between responders and non-responders (Fig. S2E, F). PD-1 expression in CD8⁺ T cells from the background liver tissues also did not differ by etiology, including NBNC hepatitis (Fig. S2G). In addition, PD-1 expression in CD8⁺ T cells from the background liver tissues was not related to PFS as well as OS by Atez/Bev therapy across etiology including NBNC hepatitis (Fig. S2H, I). Similarly, PD-1 expression in CD8⁺ T cells of the peripheral blood was not predictive of treatment efficacy and was comparable between responders and non-responders as well (Fig. S2J, K). These findings are inconsistent with a previous report focusing on PD-1^+^CD8^+^ T cells from the background liver tissues in NBNC hepatitis [13], but suggest that TIL activation is crucial for ICI efficacy in HCC across the background etiology.

### BCAAs are crucial for CD8^+^ T cell differentiation and activation

**Fig. 2:**
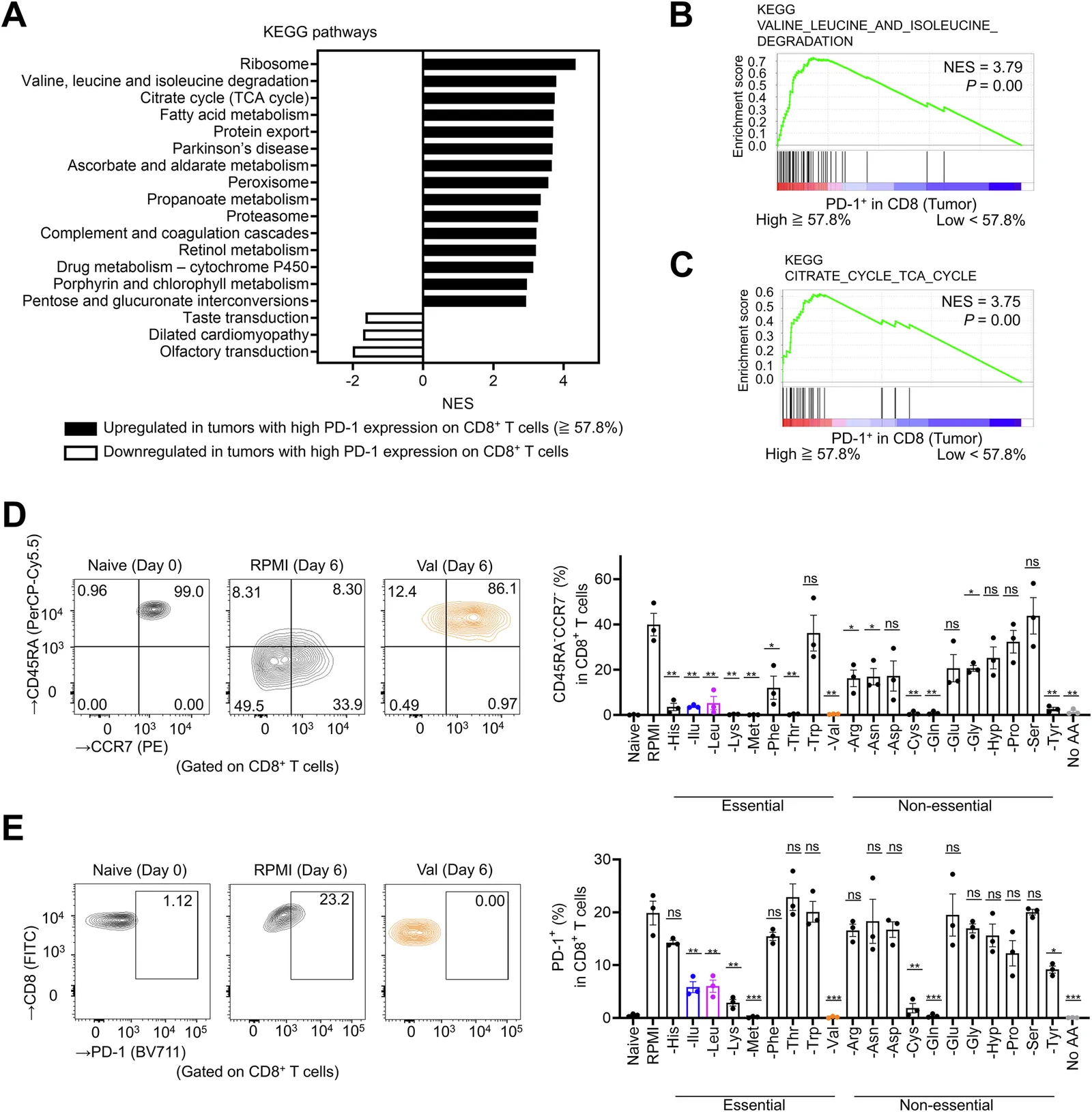
Contribution of amino acids to T-cell differentiation and activation. **A**–**C** Gene set enrichment analysis (GSEA) of the tumor samples analyzed with both RNA sequencing and flow cytometry (FCM). The samples were divided into two groups based on PD-1 expression in tumor-infiltrating CD8⁺ T cells, using a cutoff of 57.8% from the receiver operating characteristic curve in Fig. 1B, and GSEA was conducted. The top 15 significantly enriched gene sets (**A**) and GSEA plots of the valine, leucine, and isoleucine degradation pathway (**B**) and the citrate cycle (TCA cycle) pathway (**C**) are shown. CD45RA^−^CCR7^−^ effector memory subset (**D**) and PD-1 expression (**E**) in CD8^+^ T cells after chronic stimulation. Chronic stimulation was performed as described in Fig. S4A and CD8^+^ T cells were analyzed by FCM. Representative FCM staining (left) and summaries (right) are shown. All in vitro experiments were performed in triplicates. Unpaired two-tailed Student’s *t*-tests were used in (**D**, **E**) for statistical analyses. His histidine, Ilu isoleucine, Leu leucine; Lys lysine, Met methionine, Phe phenylalanine, Thr threonine, Trp tryptophan, Val valine, Arg arginine, Asn asparagine, Asp aspartic acid, Cys cysteine, Gln glutamine, Glu glutamic acid, Gly glycine, Hyp hydroxyproline, Pro proline, Ser serine, Tyr tyrosine, AA amino acid, MFI mean fluorescence intensity, ns not significant; ^*^P < 0.05; ^**^P < 0.01; ^***^P < 0.001; bar: mean; error bar: SEM.

We also performed RNA-seq on tumor tissues and compared the gene expression between two groups divided by PD-1 expression in CD8⁺ TILs. We conducted GSEA using the Kyoto Encyclopedia of Genes and Genomes (KEGG) dataset [28]. In the analysis, several AA-related metabolic pathways were found to be enriched in the group with high PD-1 expression in CD8⁺ TILs (Tables S4 and S5). Notably, BCAA-related metabolic pathway was the top-ranked among these significantly enriched AA-related metabolic pathways (Fig. 2A, B and Table S4). In addition, citrate cycle-related pathway was ranked highly among the significantly enriched pathways in the group with high PD-1 expression in CD8⁺ TILs (Fig. 2A, C and Table S4). Consistently, the BCAA-related metabolic pathway was the top-ranked among the significantly enriched AA-related metabolic pathways in the comparison between responders and non-responders to Atez/Bev therapy (Fig. S3A, B, and Tables S6, S7). Additionally, mitochondrial metabolic pathways were also significantly enriched and highly ranked in responders (Fig. S3A, C, D, and Table S6). To validate these findings in an independent cohort, we analyzed RNA-seq data of the IMbrave150 cohort, which included 181 HCC patients treated with Atez/Bev therapy [17]. Consistent with our cohort, the BCAA-related metabolic pathway was also significantly enriched in responders, defined as patients with PFS of 6 months or longer (Fig. S3E). Given these results, the BCAA’s metabolic environment of the liver tumor was considered to be important for therapeutic efficacy.

Though there are several reports suggesting the importance of AAs for T cell differentiation and activation [29], none have systematically evaluated the contribution of each individual AA. To address this, we compared differentiation and activation during chronic antigen stimulation. That is, we repeatedly stimulated naive CD8^+^ T cells with anti-CD3/28 mAbs in normal RPMI medium, 20 individual AA-deprived RPMI medium, or all AA-deprived RPMI medium (Fig. S4A) [21]. We compared CD45RA^-^CCR7^-^ effector memory subset and revealed that most of essential AAs, including BCAAs, and several non-essential AAs were necessary for effector memory differentiation (Fig. 2D). We also found that BCAAs, lysine, methionine, cysteine, glutamine, and tyrosine are critical for activation which was evaluated with PD-1 expression (Fig. 2E).

**Fig. 3:**
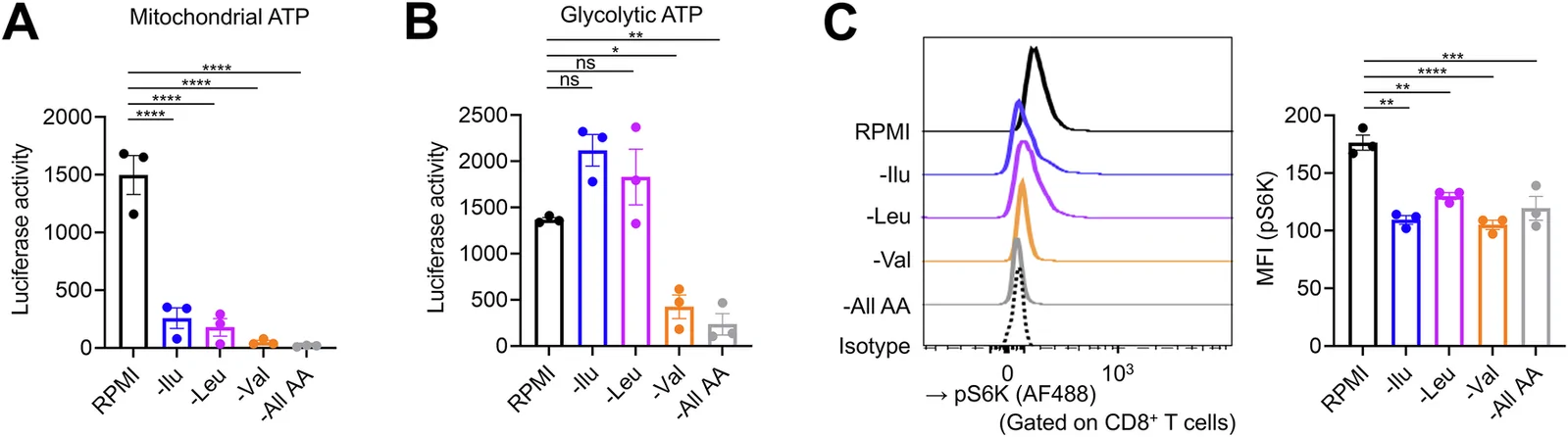
Branched chain amino acids and T cell mitochondrial function. Mitochondrial ATP production (**A**) and glycolytic ATP production (**B**) in CD8^+^ T cells after chronic stimulation. Chronic stimulation was performed as described in Figure S4A. ATP production was evaluated by luciferase activity and summaries are shown. **C** Phosphorylated S6K (pS6K) in CD8^+^ T cells after chronic stimulation. Chronic stimulation was performed as described in Fig. S4A, and CD8^+^ T cells were analyzed by flow cytometry (FCM). Representative FCM staining (left) and summary (right) are shown. All in vitro experiments were performed in triplicates. One-way analysis of variance with the Bonferroni correction was used in (**A–C**) for statistical analyses. Ilu isoleucine, Leu leucine, Val valine, AA amino acid, MFI mean fluorescence intensity, ns not significant; ^*^P < 0.05; ^**^P < 0.01; ^***^P < 0.001; ^****^P < 0.0001; bar: mean; error bar: SEM.

Given that BCAAs were crucial for CD8^+^ T cell differentiation and activation and Atez/Bev efficacy in HCC, we focused on BCAAs to evaluate their impact on CD8⁺ T cell metabolism. We repeatedly stimulated naive CD8^+^ T cells in normal RPMI medium, each BCAA-deprived medium and all AA-deprived medium as in Fig. S4B and evaluated ATP production from mitochondrial metabolism and glycolysis. As a result, the deprivation of each BCAA significantly suppressed mitochondrial ATP, suggesting the critical role of BCAAs in mitochondrial metabolism (Fig. 3A). Conversely, glycolytic ATP was significantly inhibited only by valine deprivation (Fig. 3B). Because the mTORC1 signaling pathways, which are sensitive to various metabolites, play a crucial role in T cell differentiation and activation [30], we evaluated the phosphorylation of S6K, downstream targets of mTORC1 under the same conditions. Consequently, the deprivation of each BCAA significantly suppressed the phosphorylation of S6K (Fig. 3C). Then, we stimulated the upstream pathway of mTORC1 signaling with rhIGF-I in each BCAA-deprived culture condition to assess the contribution of mTORC1 signaling to each BCAA-dependent mitochondrial metabolism (Fig. S5A) [31, 32]. As a result, leucine-dependent mitochondrial metabolism was almost completely restored with rhIGF-I, whereas isoleucine- and valine-dependent mitochondrial metabolism was partially recovered (Fig. S5B). In addition, valine-dependent glycolytic ATP production was not restored by rhIGF-I (Fig. S5C). These findings suggest that BCAAs support CD8⁺ T cell differentiation and activation mainly through mitochondrial metabolism, whereas isoleucine and valine may also act through mTORC1-independent mechanisms.

### BCAA transaminase 1 (BCAT1) in cancer cells promotes BCAA consumption, leading to metabolic competition between cancer cells and T cells

As well as T cells, cancer cells can also utilize BCAAs for proliferation and survival [33]. In addition, liver tissue, which receives absorbed BCAAs first through the portal vein, is considered to be rich in BCAAs because hepatocytes do not catabolize them due to low *BCAT1/2* expression [34, 35]. Consistently, we confirmed high BCAA concentration in liver tissues from mouse models (Fig. S6A). Thus, we hypothesized that there could be metabolic competition for BCAAs between cancer cells and CD8^+^ T cells in the hepatic TME.

To evaluate the potential compensatory relationship between *BCAT1* and *BCAT2*, we assessed their gene expression levels in Hepa1-6, MC-38, and EMT6 cell lines. As a result, we found no significant correlation between *Bcat1* and *Bcat2* expression (Fig. S6B), suggesting limited compensatory regulation between them. Because the murine HCC cell line Hepa1-6 expressed low levels of both *Bcat1* and *Bcat2*, we generated cells overexpressing either *Bcat1* or *Bcat2* to compare their roles in BCAA metabolism (Fig. S6C, D). In these overexpression models, each gene was selectively upregulated without altering the expression of the other isoform (Fig. S6C, D). Although cell proliferation was not significantly altered (Fig. S6E), *Bcat1* overexpression significantly increased BCAA consumption compared with control, while *Bcat2*-overexpression showed a similar but non-significant trend (Fig. S6F). In addition, when CD8⁺ T cells were cocultured with each cell line, effector memory differentiation and PD-1 upregulation were suppressed only under the *Bcat1*-overexpressing condition compared to control coculture (Fig. S6G, H). Consistently, production of interferon-γ (IFN-γ) and granzyme B by CD8⁺ T cells was suppressed only in coculture with *Bcat1*-overexpressing cells (Fig. S6I, J). These suppressive effects induced by *Bcat1* overexpression were restored by BCAA supplementation (Fig. S6G–J). We also compared *BCAT1* and *BCAT2* expression in tumors from our ICI-treated cohort and found no significant correlation between them (Fig. S6K). In addition, although not statistically significant, high *BCAT1* expression tended to be associated with poorer prognosis (Fig. S6L, M), whereas no such trend was observed for *BCAT2* (Fig. S6N, O). Taken together with that *BCAT1* expression was reportedly a prognostic factor in HCC [36], BCAT1 was considered to play an important role in the metabolic competition for BCAAs between cancer cells and CD8⁺ T cells.

### *BCAT1* overexpression in cancer cells induces metabolic competition for BCAAs between cancer cells and T cells in the hepatic TME and suppresses antitumor immunity in liver

**Fig. 4:**
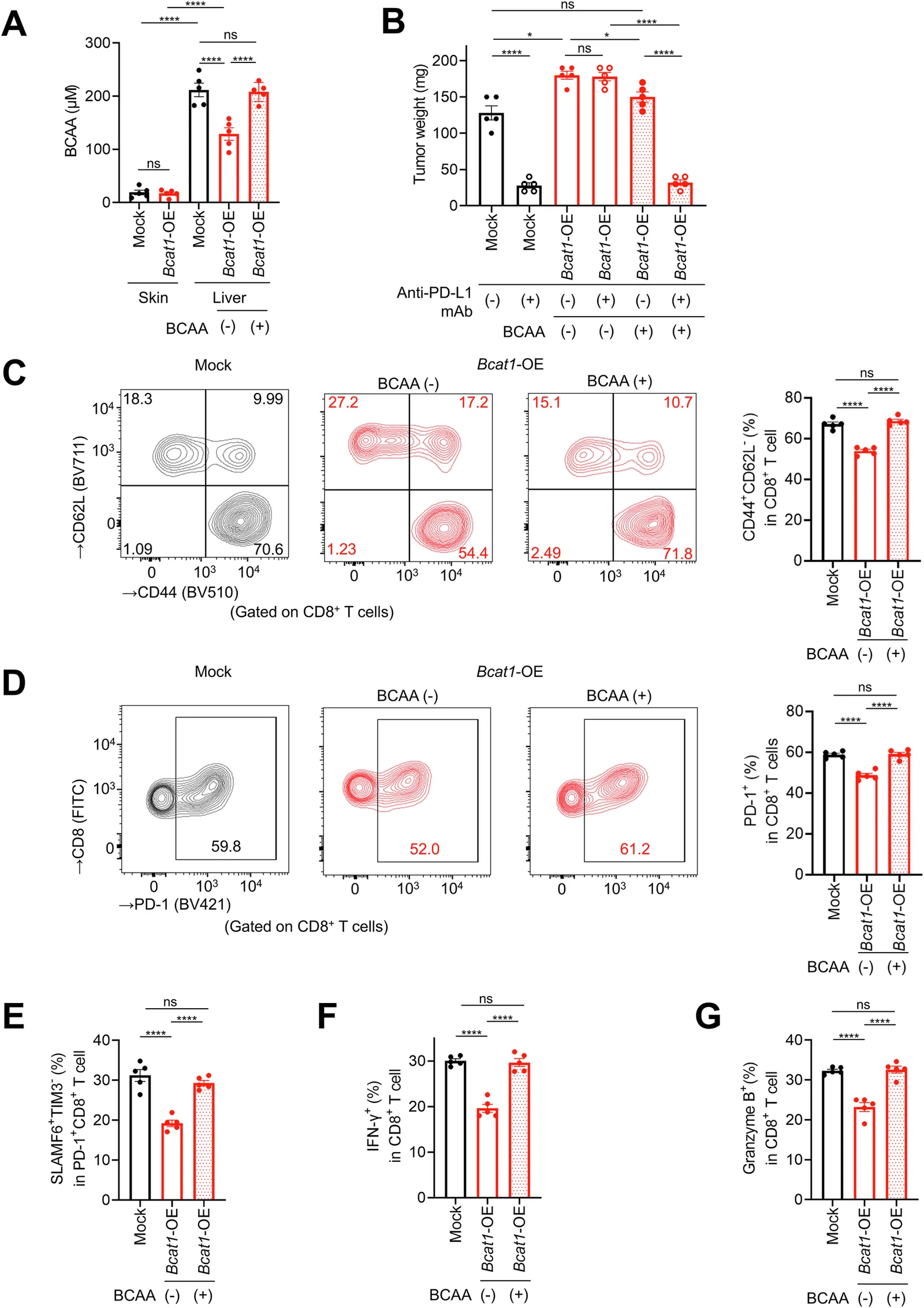
Effects of *Bcat*1 overexpression in Hepa1-6 on antitumor immunity in intrahepatic tumors. **A** Branched chain amino acid (BCAA) concentration of the subcutaneous (left) and intrahepatic (right) Hepa1-6 tumors. Tumor cells (1 × 10^6^) were inoculated subcutaneously or intrahepatically on day 0 and BCAA concentrations were evaluated on day 12. A BCAA-supplemented group was additionally included for *Bcat1*-overexpressing (OE) intrahepatic tumors. The concentrations were analyzed by colorimetric assays and summary is shown. **B** In vivo efficacy of PD-L1 blockade against intrahepatic Hepa1-6 tumors. Tumor cells (1 × 10^6^) were inoculated in a liver on day 0. Each treatment was administered on day 5, 8 and 11 (*n* = 5 per group). Tumor weight was evaluated on day 12. A BCAA-supplemented group was additionally included for *Bcat1*-OE tumors. Summary is shown. The frequencies of CD44^+^CD62L^−^ effector memory CD8^+^ T cells (**C**) and PD-1^+^CD8^+^ T cells (**D**) in tumor infiltrating lymphocytes (TILs) of the intrahepatic tumors. Tumors were harvested on day 12 to collect TILs for flow cytometry (FCM) evaluation. A BCAA-supplemented group was additionally included for *Bcat1*-OE tumors. Representative FCM staining (left) and summaries (right) are shown. The frequencies of SLAMF6^+^TIM3^−^ progenitor exhausted subset in PD-1^+^CD8^+^ TILs (**E**), and interferon-γ (IFN-γ)^+^CD8^+^ T cells (**F**) and granzyme B^+^CD8^+^ T cells (**G**) in TILs of the intrahepatic tumors. In vivo experiments were performed as described in (**C**) and (**D**). Summaries are shown. All in vivo experiments were performed in duplicate with similar results. One-way analysis of variance with Bonferroni corrections was used in (**A**–**G**) for statistical analyses. ns: not significant; ^*^P < 0.05; ^****^P < 0.0001; bar: mean; error bar: SEM.

We next evaluated the effects of *Bcat1* expression in cancer cells on antitumor immune responses in vivo using the Hepa1-6 cell lines. In the subcutaneous model, intratumoral BCAA levels were low and did not differ significantly between control and *Bcat1*-overexpressing tumors (Fig. 4A). Consistent with this, *Bcat1*-overexpressing tumors exhibited comparable growth to control tumors and responded similarly to anti-PD-L1 mAb (Fig. S7A). Conversely, in BCAA-rich intrahepatic model, *Bcat1*-overexpressing tumors exhibited reduced BCAA levels compared with control tumors, which were restored by BCAA supplementation (Fig. 4A). Consistently, control tumors showed a significant response to anti-PD-L1 mAb, whereas *Bcat1*-overexpressing tumors exhibited resistance to the treatment, which was reversed by BCAA supplementation (Fig. 4B). In addition, we assessed effector memory differentiation and activation of CD8⁺ TILs based on the CD62L^−^CD44⁺ effector memory subset and PD-1 expression. Along with the tumor growth, *Bcat1*-overexpressing tumors reduced the effector memory subset and PD-1 expression in tumor-infiltrating CD8^+^ T cells, not in the subcutaneous model but in the intrahepatic model (Figs. 4C, D and S7B, C). *Bcat1*-overexpressing tumors also reduced the progenitor exhaustion defined as SLAMF6^+^TIM3^−^ fraction among PD-1⁺CD8⁺ T cells, and impaired IFN-γ and granzyme B production by tumor-infiltrating CD8⁺ T cells, only in the intrahepatic model (Figs. 4E–G and S7D–F). The immunosuppressive intrahepatic TME induced by *Bcat1* overexpression was reversed upon BCAA supplementation (Fig. 4C–G).

In addition to Hepa1-6 cells, we evaluated the effects of BCAT1 in antitumor immunity using a *Bcat1*-overexpressing EMT6 cell line (Fig. S8A), which has low endogenous *Bcat1* expression (Fig. S6B). As observed in Hepa1-6 cells, *Bcat1* overexpression did not affect *Bcat2* expression in EMT6 cells (Fig. S8B). While *Bcat1* overexpression did not significantly affect the proliferation in vitro (Fig. S8C), it significantly increased BCAA consumption (Fig. S8D). In vivo, *Bcat1* overexpression induced BCAA depletion in the BCAA-rich liver TME, but not in subcutaneous TME (Fig. S8E). Consistently, *Bcat1* overexpression did not affect tumor growth or anti-PD-L1 mAb response in the subcutaneous model (Fig. S9A). Similarly, it had no effect on effector memory differentiation or PD-1 expression in tumor-infiltrating CD8⁺ T cells (Fig. S9B, C). In contrast, *Bcat1* overexpression conferred resistance to anti-PD-L1 mAb and significantly suppressed effector memory differentiation and PD-1 expression in tumor-infiltrating CD8⁺ T cells in the BCAA-rich intrahepatic model (Fig. S10A–C).

### *BCAT1* knockdown reduces metabolic competition for BCAAs between cancer cells and T cells in the hepatic TME

**Fig. 5:**
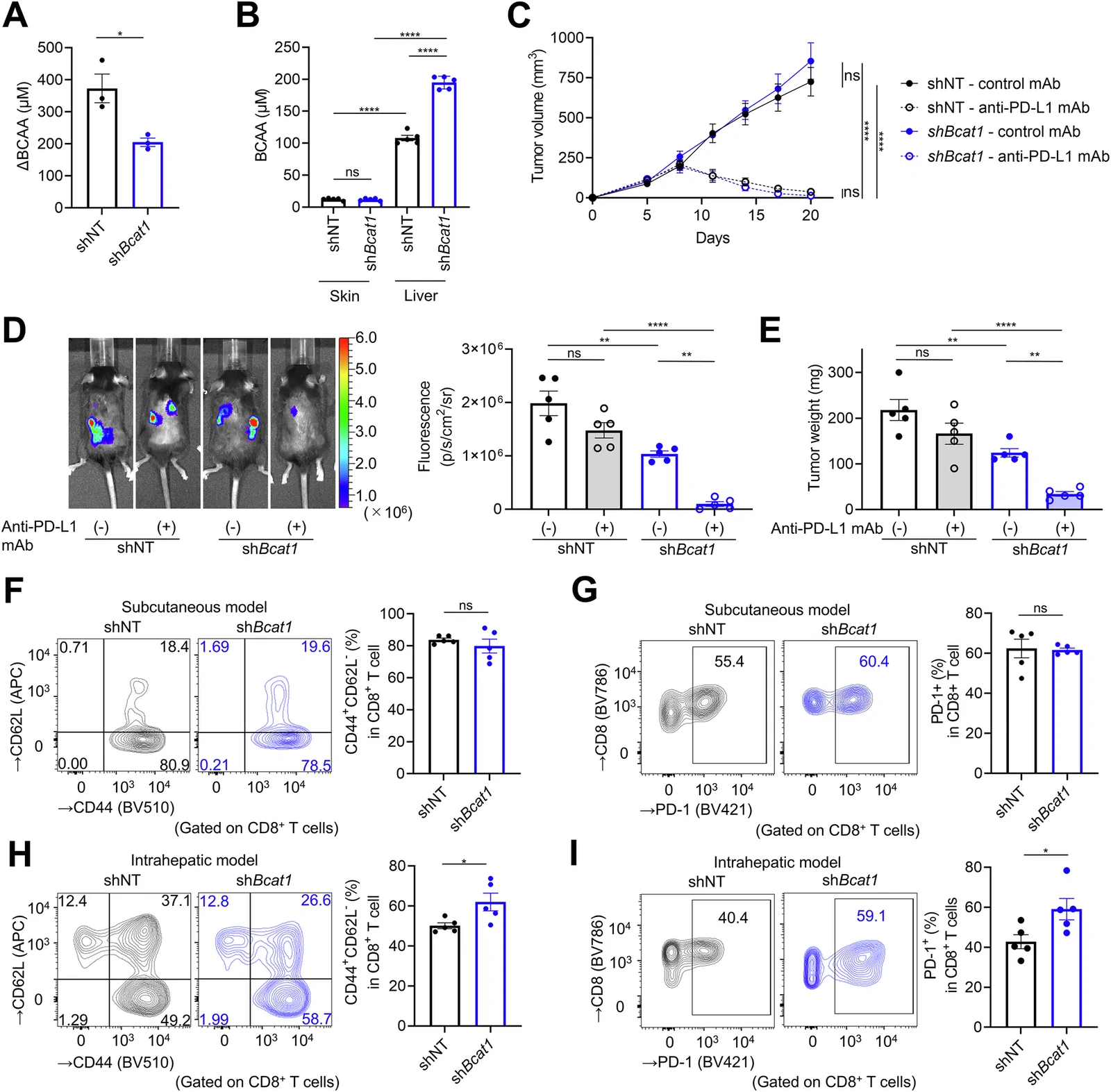
*Bcat1* knockdown in MC-38 and antitumor immunity. **A** Branched chain amino acid (BCAA) consumption. 2 × 10^5^ cells were cultured for 48 h and the BCAA concentrations in each medium were measured by colorimetric assays. BCAA consumption was assessed by calculating the difference in BCAA concentrations between the culture medium before and after incubation. Summary is shown. **B** BCAA concentration of the subcutaneous (left) and intrahepatic (right) MC-38 tumors. Tumor cells (1 × 10^6^) were inoculated subcutaneously or intrahepatically on day 0 and BCAA concentration was evaluated on day 12. The concentrations were analyzed by colorimetric assays and summary is shown. **C** In vivo efficacy of anti-PD-L1 monoclonal antibody (mAb) against subcutaneous MC-38 tumors. Tumor cells (1 × 10^6^) were inoculated subcutaneously on day 0. Each treatment was administered on days 5, 8, and 11 (*n* = 5 per group). Summary of tumor curves is shown. **D**, **E** In vivo efficacy of anti-PD-L1 mAb against intrahepatic MC-38 tumors. Tumor cells (1 × 10^6^) were inoculated in a liver on day 0. Each treatment was administered on days 5, 8, and 11. Intrahepatic tumor volume with bioluminescence signals using luciferase-transduced cells (**D**) and tumor weight (**E**) were evaluated on day 12. Representative imaging (**D**, left) and summaries (**D**, right; **E**) are shown. The frequencies of CD44^+^CD62L^−^CD8^+^ effector memory T cells (**F**) and PD-1^+^CD8^+^ T cells (**G**) in tumor infiltrating lymphocytes (TILs) of the subcutaneous tumors. Tumors were harvested on day 7 to collect TILs for flow cytometric (FCM) evaluation. Representative FCM staining (left) and summaries (right) are shown. The frequencies of CD44^+^CD62L^−^ effector memory CD8^+^ T cells (**H**), PD-1^+^CD8^+^ T cells (**I**) in TILs of the intrahepatic tumors. Tumors were harvested on day 14 to collect TILs for FCM evaluation. Representative FCM staining (left) and summaries (right) are shown. All in vitro experiments were performed in triplicates and all in vivo experiments were performed in duplicate, with similar results. Unpaired two-tailed Student’s *t*-tests were used in (**A**) and (**F**–**I**), one-way analysis of variance (ANOVA) with Bonferroni corrections was used in (**B**, **D**, **E**), and two-way ANOVA with the Bonferroni correction was used in (**C**) for statistical analyses. shNT: nontargeting short hairpin RNA; sh*Bcat1*: *Bcat1* short hairpin RNA; ns: not significant; ^*^P < 0.05; ^**^P < 0.01; ^****^P < 0.0001; bar: mean; error bar: SEM.

Because *Bcat1* expression was high in MC-38 cells (Fig. S6B), we created a *Bcat1*-knockdown MC-38 cell line using shRNA, which did not affect *Bcat2* expression (Fig. S11A, B). While *Bcat1* knockdown did not significantly affect the proliferation in vitro (Fig. S11C), it suppressed BCAA consumption (Fig. 5A). In vivo, *Bcat1* knockdown rescued BCAA depletion only in the BCAA-rich intrahepatic model (Fig. 5B). Consistently, *Bcat1* knockdown did not affect tumor growth or anti-PD-L1 mAb response in the subcutaneous model (Fig. 5C), whereas it slowed tumor progression and enhanced response to anti-PD-L1 mAb treatment in the intrahepatic model (Fig. 5D, E). In addition, CD8⁺ TILs in *Bcat1*-knockdown tumors had significantly more effector memory population and higher PD-1 expression compared to those in control tumors only in the intrahepatic model (Fig. 5F–I). Along with the results from *Bcat1* overexpression experiments, these indicate that BCAT1-mediated metabolic competition for BCAA between cancer cells and TILs in the BCAA-rich hepatic TME contributes to suppression of antitumor immunity.

### High expression of *BCAT1* is associated with poor prognosis in HCC

Considering the results of our experiments, we also analyzed the relationship between *BCAT1* expression and patient prognosis using publicly available datasets (TCGA, Firehose Legacy) obtained from cBioPortal [19, 20]. In accordance with the organs in which BCAA levels were measured in mice, we compared prognoses of the HCC, melanoma, lung cancer and kidney cancer cohorts, respectively, according to *BCAT1* expression. For each cancer type, patients were stratified into high and low-*BCAT1* expression groups based on the median *BCAT1* expression, and OS was compared using Kaplan–Meier curves and the log-rank test. As a result, the OS was significantly shorter in the *BCAT1*-high expression group only in HCC (Fig. S12A–E). Altogether, BCAA metabolism is important particularly for the BCAA-rich hepatic TME.

## Discussion

Cancer immunotherapies targeting PD-1/L1 and/or CTLA-4 have become the standard treatment for advanced HCC [3, 37, 38]. However, the response rate remains below 50%, necessitating further elucidation of the mechanisms underlying response and resistance to identify better biomarkers and develop strategies to overcome resistance. Tumor mutational burden (TMB), which reflects the number of somatic mutations, has been proposed as a predictive biomarker for ICI therapy and is utilized for prognostication in several cancer types [39]. However, in HCC, TMB reportedly does not correlate with prognosis [40]. While PD-L1 expression has been widely evaluated as another biomarker candidate, its predictive value has been insufficient in various cancer types including HCC [41, 42]. Given these limitations, which highlight the need for disease-specific investigations using clinical specimens, we analyzed clinical samples from HCC patients and demonstrated that PD-1^+^CD8^+^ T cells in the TME could serve as a potential biomarker for ICI efficacy. In addition, this study also proved the importance of BCAA metabolism in TIL activation, specifically within the BCAA-rich hepatic TME and ICI efficacy against liver tumors. Notably, while previous studies have suggested the contribution of BCAAs to CD8⁺ T-cell function [24, 43], this study provides the first evidence of liver-specific metabolic competition for BCAAs based on analyses of clinical specimens from multiple cohorts as well as various experimental models.

We analyzed 50 cases treated with Atez/Bev combination therapy and found that etiology, including viral hepatitis and NBNC hepatitis, was not associated with prognosis. The TIL analyses demonstrated that TIL activation, as evaluated by PD-1 expression in CD8^+^ T cells, plays a crucial role in ICI response in HCC, consistent with previous studies including ours [25, 26]. While a previous study reported that MASLD leads to the accumulation of PD-1^+^CD8^+^ T cells, potentially promoting hepatitis and tumor progression upon ICI treatment, resulting in poor prognosis [13], the results were validated using only mouse models. In contrast, the upregulation of PD-1 expression in CD8^+^ T cells within the background liver tissues of NBNC hepatitis was not distinctive and was comparable to that of other etiologies from our present study. In addition, PD-1 expression in CD8^+^ T cells of the background liver tissues was not correlated with ICI prognosis. Although we evaluated not only MASLD but also other non-virus etiologies with small sample size, these findings suggest the limitation of mouse model and the need to analyze clinical samples.

We demonstrated that BCAA metabolism played important roles in antitumor immunity based on both clinical sample analyses of multi-cohorts and experimental results. Various AAs are reportedly important for T cell effector memory differentiation and activation [29] and our GSEA based on KEGG dataset, which includes numerous AA-related metabolic pathways, revealed that several AA-related metabolic pathways were enriched in the group with high PD-1 expression in CD8⁺ TILs and in ICI-responders. Systematic validation with our unique experimental system further suggested that not only BCAAs but also several other AAs could also contribute to T cell effector memory differentiation and activation. Notably, however, BCAA-related metabolic pathway consistently ranked highest in our GSEA, underscoring their predominant role. In addition, consistent with previous reports [44, 45], we demonstrated through our unique experimental system that BCAAs activate both mTORC1 signaling and mitochondrial metabolism in T cells, thereby promoting their differentiation and activation. Although IGF-I restored mitochondrial metabolism in T cells under leucine-depleted condition by activating upstream pathway of mTORC1, it did not fully restore mitochondrial metabolism under isoleucine- or valine-depleted conditions. In addition, only valine contributed to glycolytic activity in our experimental model. Intact BCAAs, not their downstream metabolites, reportedly regulate both glycolytic and mitochondrial programs through mTOR and FOXO1 signaling [24]. Although leucine is known to regulate mitochondrial metabolism through mTORC1 activation [46, 47], it has also been reported that intact isoleucine and valine maintain c-Myc expression independently of BCAA catabolism, which support cellular metabolism including glycolysis [48, 49]. Considering our experimental findings and these previous reports, individual BCAAs may differentially influence T-cell metabolism, differentiation, and activation through distinct signaling contributions. The distinct roles of individual BCAAs are not fully clarified, necessitating further investigation.

Because BCAAs are little degraded in the liver after supplied orally and enter the portal system [50], BCAAs are rich in the liver tissues as shown in our murine analyses. Altogether, our results showing the importance of BCAA metabolism in liver-specific antitumor immunity and liver-specific BCAA competition could be reasonable. Given that both *BCAT1/2* regulate the first key step in BCAA metabolism, including both degradation and synthesis [51], we investigated whether each enzyme compensates the expression level of each other. As a result, neither significant correlation nor compensatory relationship between *BCAT1* and *BCAT2* expression was observed. While hepatocytes exhibit minimal expression of *BCAT1/2*, HCC reportedly upregulates *BCAT1* during progression, contributing to tumor proliferation and chemotherapy resistance [36, 52]. Although a previous report has suggested a synthetic role in hematologic malignancies [53], we demonstrated that *BCAT1* predominantly facilitates BCAA degradation in solid tumors including HCC as mentioned in other previous studies [33, 36, 54, 55]. Although both *BCAT1* and *BCAT2* tended to contribute to BCAA degradation in an HCC cell line, the effect was significant only for *BCAT1* in our experimental setting, suggesting a more predominant role for *BCAT1*. In addition, high *Bcat1* expression in cancer cells suppressed antitumor immune responses not in the subcutaneous but in the BCAA-rich intrahepatic model in vivo, which was reversed by BCAA supplementation. Furthermore, *Bcat1* overexpression induced immunosuppression specifically in intrahepatic tumors by impairing CD8⁺ T-cell activation, effector differentiation, and production of cytokines and cytotoxic molecules. *Bcat1* overexpression was also associated with suppression of progenitor exhaustion, which represents an ICI-responsive state [56]. These immunosuppressive phenotypes induced by metabolic competition for BCAAs were recovered by BCAA supplementation in the intrahepatic model. Although multiple site-specific factors may underlie the differences between subcutaneous and intrahepatic models, the rescue by BCAA supplementation indicates that altered BCAA availability is important for antitumor immunity in the liver. Considering these findings, upregulation of *BCAT1* can benefit cancer cells by attenuating antitumor immune responses through limiting BCAA availability to TILs, particularly within the BCAA-rich hepatic TME. In addition to *BCAT1*, several previous studies have reported that high expression of *SLC7A5,* encoding LAT1, is associated with poor prognosis of HCC [57, 58]. While LAT1 also transports other AAs than BCAAs, its prognostic relevance can further support the importance of BCAA metabolism in HCC. Although the metabolic competition for glucose and several AAs including BCAAs has already been reported [8–11, 43], this is the first study to demonstrate liver-specific metabolic competition for BCAAs based on analyses of clinical specimens from multiple cohorts as well as various experimental models.

HCC typically arises from cirrhosis following chronic hepatitis. As liver cirrhosis progresses, skeletal muscle consumes BCAAs to compensate for impaired hepatic urea cycle and to detoxify ammonia, leading to reduction in systemic BCAA levels [59]. Given the importance of BCAAs in promoting antitumor immunity as demonstrated in our study, such BCAA loss can impair antitumor immunity and limit ICI efficacy. Therefore, in addition to preventing hepatic encephalopathy, muscle loss, and liver-related death [60], BCAA supplementation can reverse the suppressed antitumor immunity in cirrhotic patients as we demonstrated in our in vivo experiments. Supporting this, BCAAs are also reported to reduce the risk of hepatic carcinogenesis [60]. We consider BCAA supplementation in the pre-neoplastic stage can enhance antitumor immunity by supporting lymphocytes responsible for immune surveillance. In contrast, once HCC has developed, cancer cells may upregulate *BCAT1* to utilize BCAAs, leading to metabolic alterations favorable for HCC progression. Unfortunately, the clinical application of BCAT inhibitors has been limited due to the lack of compounds with sufficient selectivity or potency [61]. Even so, based on our findings, we propose that combining BCAT inhibitors with ICI therapy will be effective in overcoming metabolic competition for BCAAs between TILs and cancer cells, especially in tumors with high *BCAT1* expression.

Although the relevance of BCAA metabolism was supported by public RNA-seq analyses across multiple independent ICI-treated HCC cohorts, validation of fresh TIL analyses remained limited to a single cohort. While fresh TIL profiling is a powerful approach for precise evaluation of CD8⁺ T-cell activity, its routine clinical application and large-scale validation are challenging because fresh (non-frozen/fixed) tumor tissues are not routinely available. In addition, although several studies using multiplex immunohistochemistry have demonstrated that CD8⁺ T-cell activity contributes to clinical responses to ICIs [62, 63], further methodological standardization is needed before clinical implementation. Therefore, we consider that further validation in large-scale cohorts, along with the refinement and standardization of clinically feasible approaches, will be important for future clinical implementation.

In summary, we analyzed paired HCC tumor and background liver tissues from 50 patients with HCC, and demonstrated the importance of TILs, rather than lymphocytes in the background liver, in antitumor immunity across etiologies. This study also revealed that liver-specific BCAA metabolism contributed to antitumor immunity against liver tumors based on both clinical sample analysis of multi cohorts and experimental results. Our findings propose the importance of targeting BCAA metabolism as a pivotal axis to improve ICI efficacy. In addition, we highlight the significance of assessing the TME considering organ-specific metabolism, which can lead to discovery of better biomarkers and the development of metabolism-based therapies. Further studies with large cohorts are necessary to expand this study to the clinical phase.

## Supplementary information

Supplementary Figures and Tables

## Data Availability

Further information and requests for resources and reagents should be directed to and will be fulfilled by the Lead Contact, Yosuke Togashi (ytogashi1584@gmail.com). The RNA-seq dataset is publicly available at the Gene Expression Omnibus (GEO) database hosted by the National Center for Biotechnology Information (NCBI) (accession numbers: GSE285963). Clinical sample data for this study were obtained through a collaborative research project between Chiba University and Chugai Pharmaceutical Co., Ltd. All other data for this paper have been included in the main manuscript and the Supplementary data. This paper does not report the original code. The plasmids and cell lines generated in this study are available from lead contact; however, a complete material transfer agreement may be required.
