# Supplementary material for: Liver-specific amino acid metabolism impacts on efficacy of cancer immunotherapy
Source: Oncogene. 2026 Oct 17;45(46):5035–47. doi: 10.1038/s41388-026-04023-0 (PMC13723418; doi:10.1038/s41388-026-04023-0)
Supplement: Supplementary file 1 [file 41388_2026_4023_MOESM1_ESM.pdf]

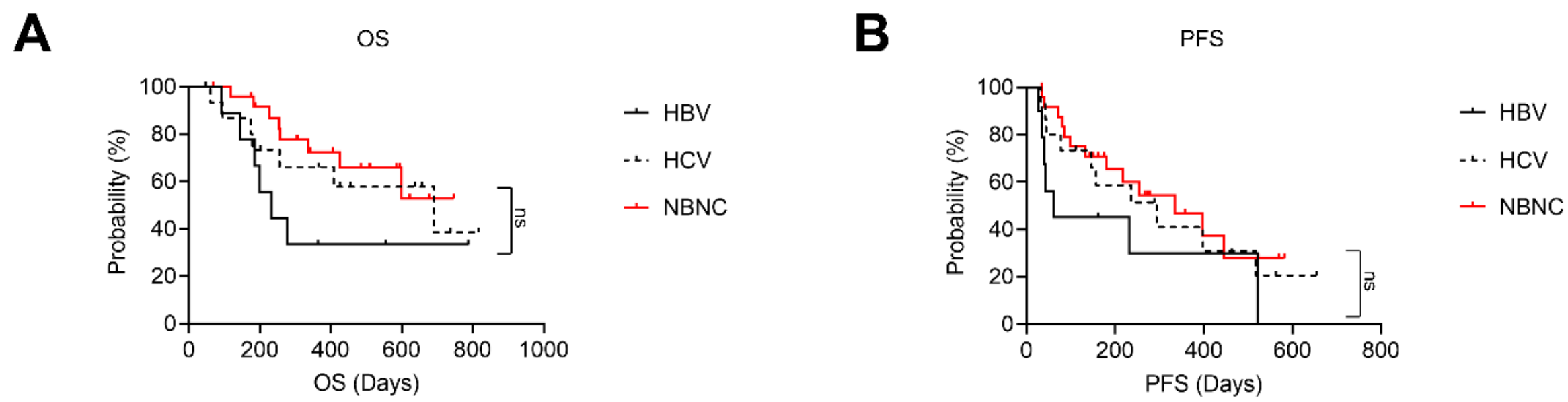

Fig. S1. Prognosis stratified by etiology in hepatocellular carcinoma (HCC)
(A and **B)** Kaplan–Meier curves for overall survival (OS) **(A)** and progression-free survival (PFS) **(B)** of HCC patients treated with combination therapy of atezolizumab and bevacizumab stratified by hepatitis B virus (HBV) (n = 10), hepatitis C virus (HCV) (n = 15) and non-B, non-C hepatitis (NBNC) (n=25). OS was defined as the time from initiation of treatment to death from any cause, and PFS was defined as the time from initiation of treatment to disease progression or death from any cause.

The log-rank test was used to compare Kaplan–Meier curves for statistical analyses. ns: not significant.

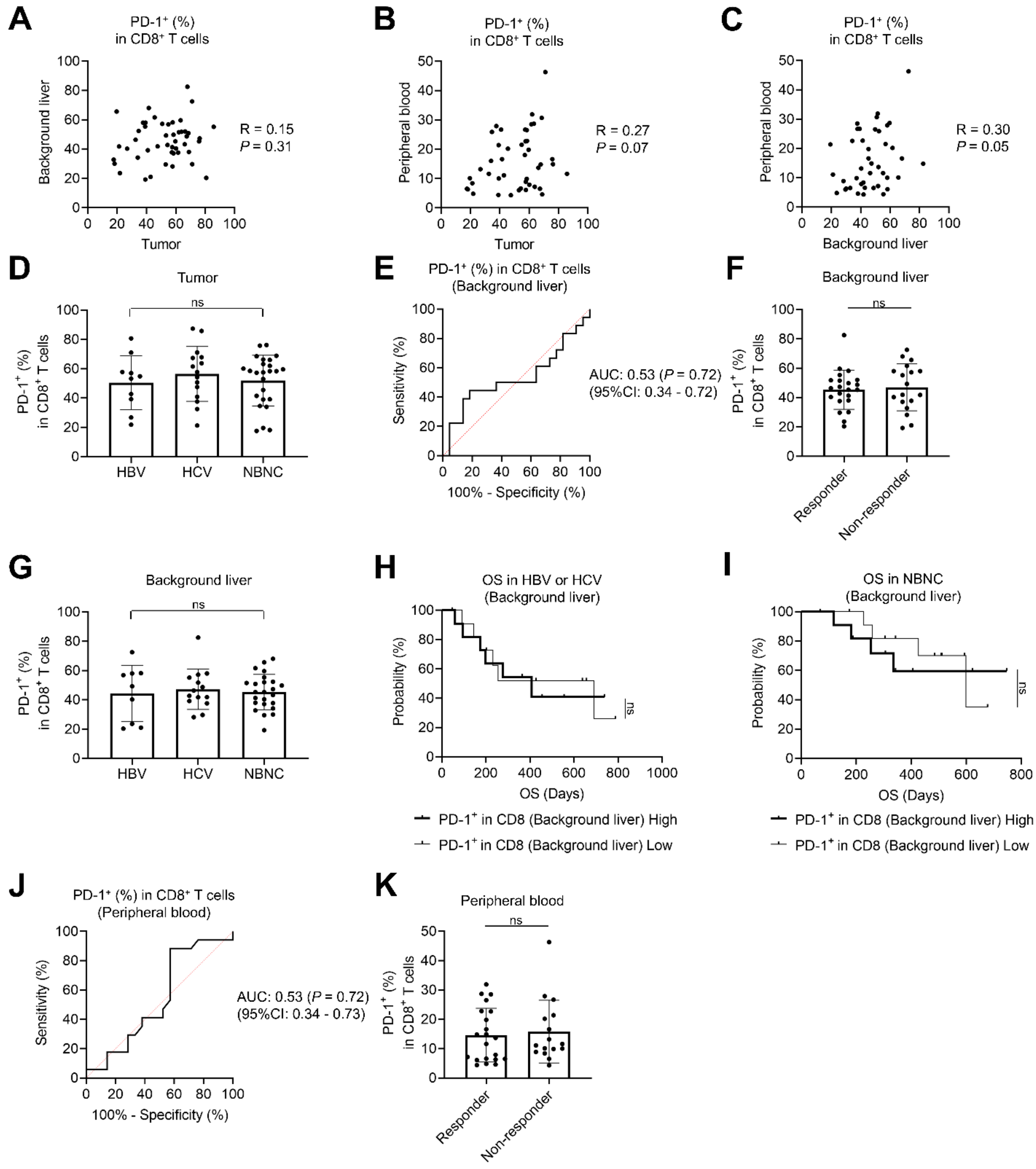

Fig. S2. Additional data of clinical samples in hepatocellular carcinoma (HCC)

Each sample was processed and analyzed as described in **Fig. 1A**. Progression-free survival (PFS) was defined as the duration from the initiation of therapy to the first documented disease progression or death from any cause, and patients whose PFS was longer than 6 months are defined as responders.

(A–C) Scatter plots illustrating the relationships of PD-1^{+} (%) in CD8^{+} T cells between the tumor tissues, background liver tissues, and peripheral blood, with Pearson's correlation coefficients (R) indicated.

(D) PD-1 expression in $CD8^+$ TILs according to different etiologies (hepatitis B virus (HBV), n = 10; hepatitis C virus (HCV), n = 15; non-B non-C hepatitis (NBNC), n = 25).
(E) Receiver operating characteristic (ROC) curve for $PD\text{-}1^+$ (%) in $CD8^+$ T cells of the background liver tissues to predict responders.
(F) PD-1 expression in $CD8^+$ T cells of the background liver tissues according to response.
(G) PD-1 expression in $CD8^+$ T cells of background liver tissues according to different etiologies (HBV, n = 9; HCV, n = 14; NBNC, n = 24).
(H and **I)** Kaplan–Meier curves for overall survival (OS) of viral hepatitis-related (n = 23) **(H)** and NBNC-related (n = 24) **(I)** HCC patients stratified by PD-1 expression in $CD8^+$ T cells of background liver. The cutoff for $PD\text{-}1^+$ (%) in $CD8^+$ T cells was determined using the median value. OS was defined as the time from initiation of therapy to death from any cause.
(J) ROC curve for $PD\text{-}1^+$ (%) in $CD8^+$ T cells of the peripheral blood to predict responders for HCC patients.
(K) PD-1 expression in $CD8^+$ T cells of the peripheral blood according to response.

One-way analysis of variance with the Bonferroni correction was used in **(D)** and **(G)**, unpaired two-tailed Student's *t*-test was used in **(F)** and **(K)**, and the log-rank test was used to compare Kaplan–Meier curves in **(H)** and **(I)** for statistical analyses. AUC: Area under the curve; ns: not significant; $^*P < 0.05$; bar: mean; error bar: SEM.

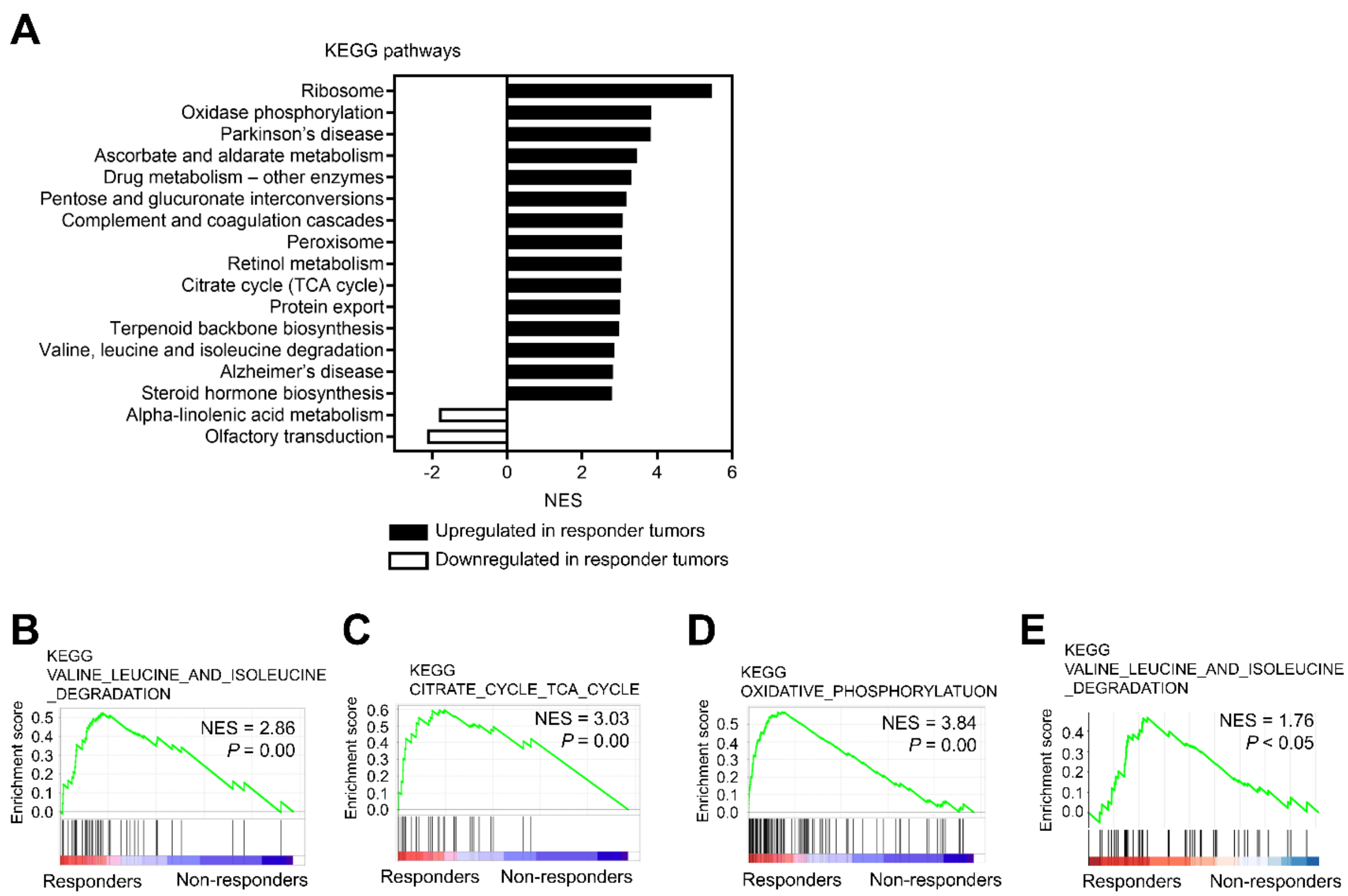

Fig. S3. Additional gene set enrichment analysis (GSEA) data

(A–D) GSEA of the tumor samples in our cohort. GSEA was conducted between responders (progression-free survival [PFS] ≥ 6 months) and non-responders. PFS was defined as the duration from the initiation of therapy to the first documented disease progression or death from any cause. The top 15 significantly enriched gene sets **(A)** and GSEA plots of the valine, leucine, and isoleucine degradation pathway **(B),** the citrate cycle (TCA cycle) pathway **(C),** and the oxidative phosphorylation pathway **(D)** are shown.

(E) GSEA plots of the valine, leucine, and isoleucine degradation pathway of the tumor samples in IMbrave150 cohort. GSEA was conducted between responders (PFS ≥ 6 months) and non-responders.

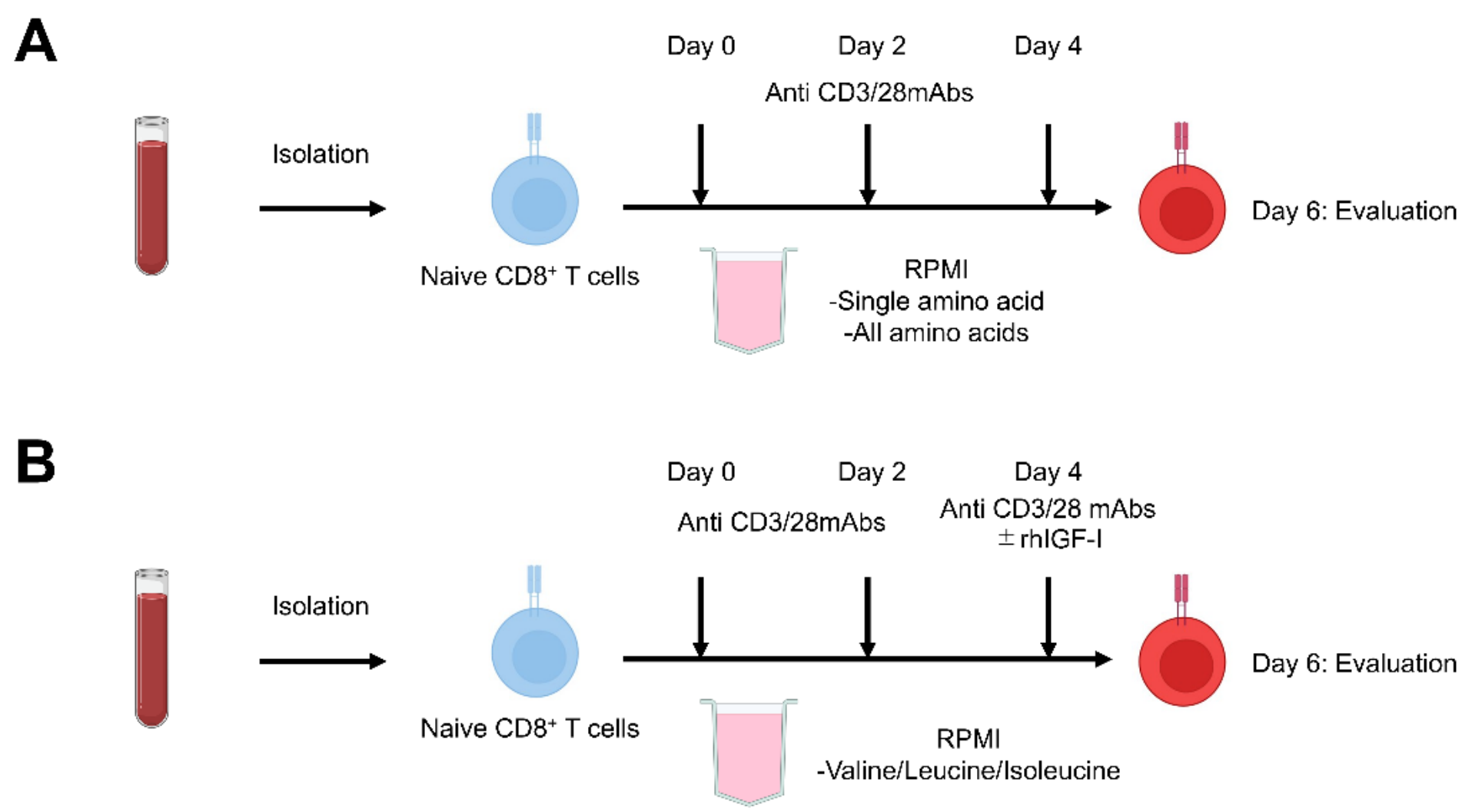

Fig. S4. Experimental schema for T cell stimulation

Isolated naive $CD8^+$ T cells were cultured in complete medium, 20 individual AA-deprived medium, or all AA-deprived medium. They were stimulated with anti-CD3/CD28 monoclonal antibody every 48 hours for 3 times to mimic chronic antigen stimulation **(A)**. To stimulate the upstream pathway of mammalian target of rapamycin complex 1 signaling, recombinant human insulin-like growth factor I (rhIGF-I) was added from the third round of anti-CD3/CD28 stimulation **(B)**. The stimulated T cells were subsequently analyzed in downstream assays.

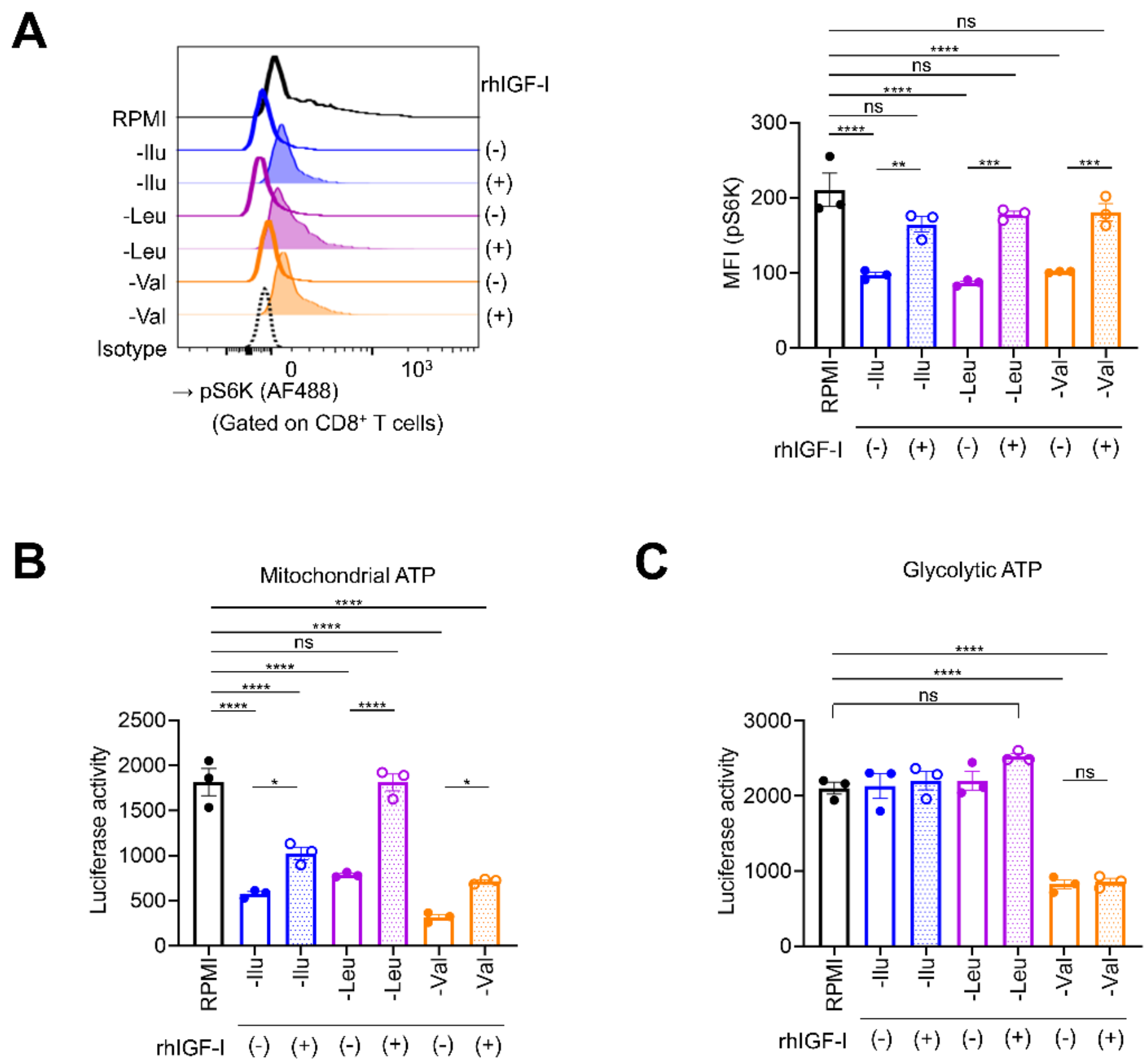

Fig. S5. Additional data related to T cell stimulation experiments

(A) Phosphorylated S6K (pS6K) in $CD8^+$ T cells after chronic stimulation with or without recombinant human insulin-like growth factor I (rhIGF-I). Chronic stimulation with or without rhIGF-I was performed as described in **Fig. S4B**, and $CD8^+$ T cells were analyzed by flow cytometry (FCM). Representative FCM staining (left) and summary (right) are shown.

(B and **C)** Mitochondrial ATP production **(B)** and glycolytic ATP production **(C)** in $CD8^+$ T cells after chronic stimulation with or without rhIGF-I. Chronic stimulation with or without rhIGF-I was performed as described in **Fig. S4B**. ATP production was evaluated by luciferase activity and summaries are shown.

All *in vitro* experiments were performed in triplicates. One-way analysis of variance with the Bonferroni correction was used in **(A–C)** for statistical analyses. Ilu: Isoleucine; Leu: Leucine; Val: Valine; AA: amino acid; MFI: Mean Fluorescence Intensity; ns: not significant; $^{*}P < 0.05$; $^{****}P < 0.0001$; bar: mean; error bar: SEM.

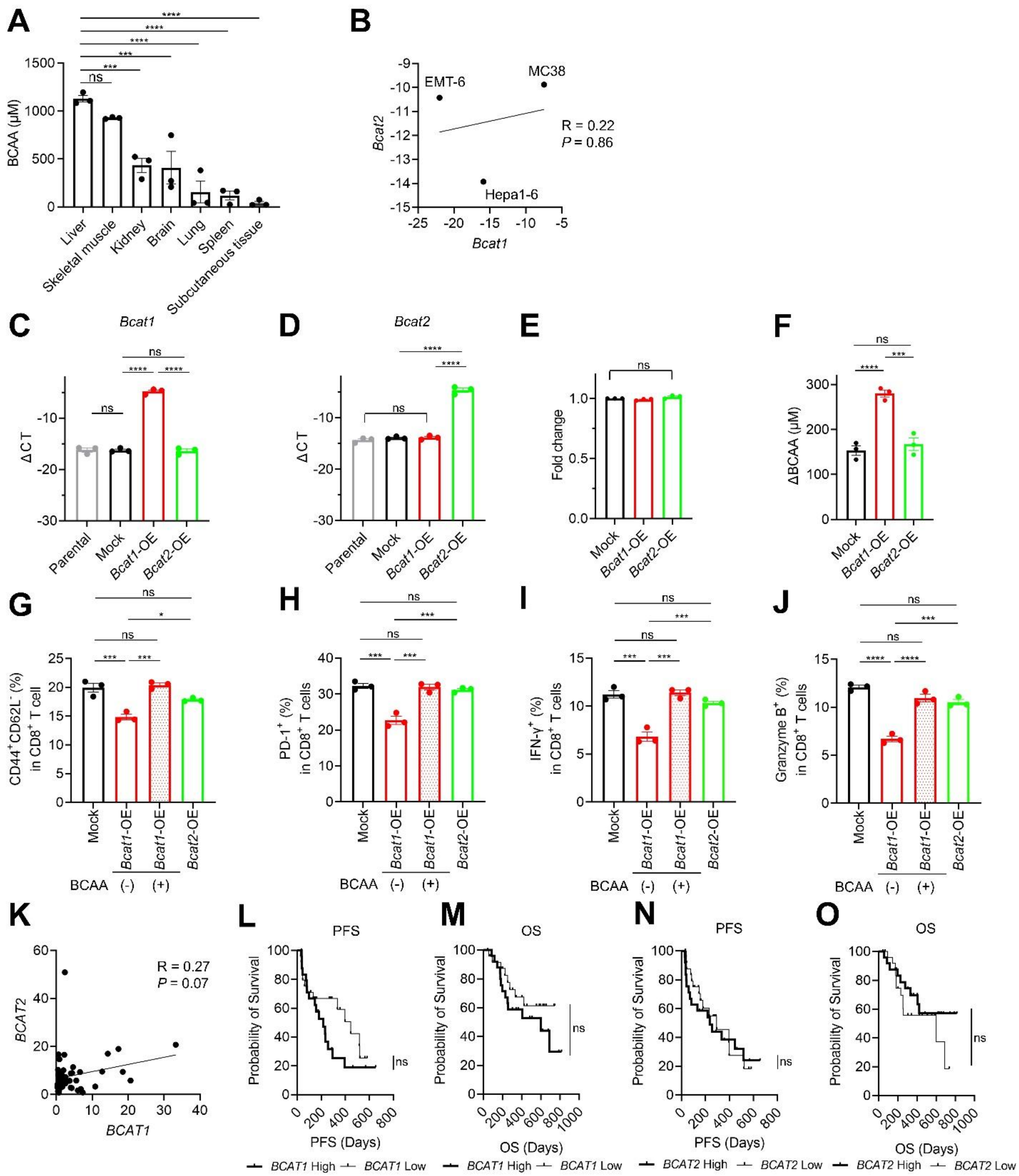

Fig. S6. Additional data related to branched chain amino acid (BCAA) metabolism and comparison between *Bcat1* and *Bcat2*

(A) BCAA concentrations of various organs in mice. The concentrations were analyzed by colorimetric assays and summary is shown.

(B) Corelation between *Bcat1* and *Bcat2* expression in murine cell lines. Gene expression was evaluated with reverse transcription quantitative polymerase chain reaction (RT-qPCR). Murine *Actb* was used as an internal control. Each Δcycle threshold (CT) was calculated by subtracting

the CT value of each gene from that of *Actb*. The mean value of three independent measurements was plotted for each cell line. Pearson's correlation coefficients (R) and P-value are shown.
(C and **D)** *Bcat1* **(C)** and *Bcat2* **(D)** expression in Hepa1-6 cell lines. Gene expression was evaluated with RT-qPCR. Murine *Actb* was used as an internal control. Each ΔCT was calculated by subtracting the CT value of each gene from that of *Actb*, and summaries are shown.
(E) Cell proliferation according to *Bcat1*/*Bcat2*-overexpression (OE). Hepa1-6 cells (2×10^4 cells) were cultured for 48 hours, followed by cell counting based on absorbance measurement. The fold change relative to mock is calculated and summary is shown.
(F) BCAA consumption. 2×10^5 cells were cultured for 48 hours and the BCAA concentrations in each medium were measured by colorimetric assays. BCAA consumption was assessed by calculating the difference in BCAA concentrations between the culture medium before and after incubation. Summary is shown.
(G–J) The frequencies of $CD44^+CD62L^-CD8^+$ effector memory T cells **(G)** and PD-1^+CD8^+ T cells **(H)**, interferon-γ (IFN-γ)$^+CD8^+$ T cells **(I)** and granzyme B $^+CD8^+$ T cells **(J)**. Isolated $CD8^+$ T cells (2×10^5) and tumor cells (2×10^5) were cocultured for 48 hours, $CD8^+$ T cells were analyzed by flow cytometry, and summaries are shown.
(K) Correlation between *BCAT1* and *BCAT2* expression levels in the HCC tumor samples treated with combination therapy of atezolizumab and bevacizumab (n = 50). Transcripts per million values derived from RNA sequencing data are plotted. Pearson's correlation coefficients (R) and P-value are shown
(L and **M)** Kaplan–Meier curves for progression-free survival (PFS) **(L)** and overall survival (OS) **(M)** of hepatocellular carcinoma (HCC) patients treated with combination therapy of atezolizumab and bevacizumab stratified by median of *BCAT1* expression (n = 50). PFS was defined as the time from initiation of treatment to disease progression or death from any cause, and OS was defined as the time from initiation of treatment to death from any cause.
(N and **O)** Kaplan–Meier curves for PFS **(N)** and OS **(O)** of HCC patients treated with combination therapy of atezolizumab and bevacizumab stratified by median of *BCAT2* expression (n = 50).

All experiments were performed in triplicates. One-way analysis of variance with the Bonferroni correction was used in **(A)** and **(C–J)**, and the log-rank test was used in **(L–O)** to compare Kaplan–Meier curves for statistical analyses. ns: not significant; $^{*}P < 0.05$; $^{***}P < 0.001$; $^{****}P < 0.0001$; bar: mean; error bar: SEM.

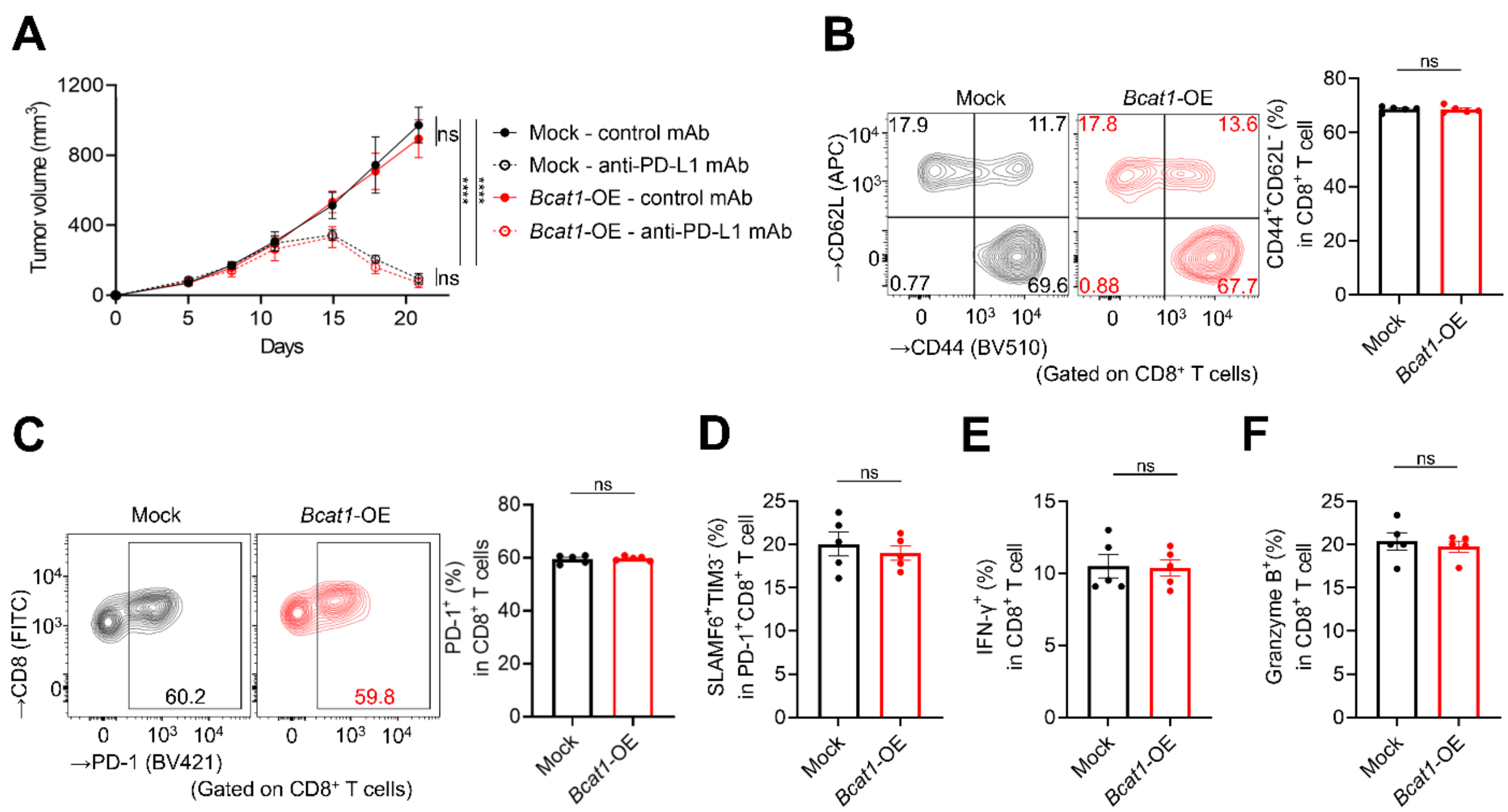

Fig. S7. Additional data related to subcutaneous Hepa1-6 tumor experiments

(A) *In vivo* efficacy of anti-PD-L1 monoclonal antibody (mAb) against subcutaneous Hepa1-6 tumors. Tumor cells (1×10^6) were inoculated subcutaneously on day 0. Each treatment was administered on day 5, 8 and 11 (n = 5 per group). Summary of tumor curves is shown.

(B and **C)** The frequencies of $CD44^+CD62L^-$ effector memory $CD8^+$ T cells **(B)** and $PD\text{-}1^+CD8^+$ T cells **(C)** in tumor infiltrating lymphocytes (TILs) of the subcutaneous tumors. Tumors were harvested on day 12 to collect TILs for flow cytometry (FCM) evaluation. Representative FCM staining (left) and summaries (right) are shown.

(D-F) The frequencies of $SLAMF6^+TIM3^-$ progenitor exhausted subset in $PD\text{-}1^+CD8^+$ TILs, and interferon-γ $(IFN\text{-}\gamma)^+CD8^+$ T cells **(E)** and granzyme B^+CD8^+ T cells **(F)** in TILs of the subcutaneous tumors. In vivo experiments were performed as described in **(B)** and **(C)**. Summaries are shown.

All *in vivo* experiments were performed in duplicate with similar results. Two-way analysis of variance with the Bonferroni correction was used in **(B)**, and unpaired two-tailed Student's *t*-tests were used in **(B–F)** for statistical analyses. OE: overexpression; ns: not significant; $^{****}P < 0.0001$; bar: mean; error bar: SEM.

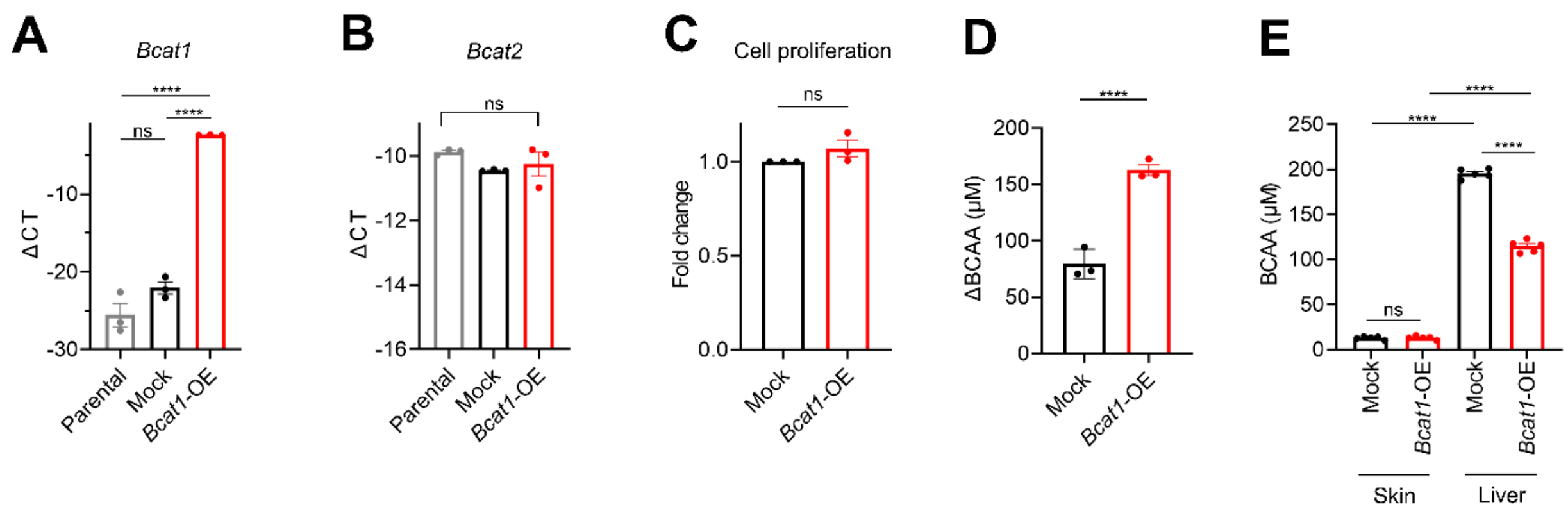

Fig. S8. *Bcat1* overexpression in EMT6 and branched chain amino acid (BCAA) metabolism
(A and **B)** *Bcat1* **(A)** and *Bcat2* **(B)** expression in *Bcat1*-overexpressing (OE) EMT6 cells. Gene expression was evaluated with reverse transcription quantitative polymerase chain reaction (RT-qPCR). Murine *Actb* was used as an internal control. Each Δcycle threshold (CT) was calculated by subtracting the CT value of each target gene from that of *Actb*, and summary is shown.
(C) Cell proliferation according to *Bcat1*-overexpression. EMT6 cells (2×10^4 cells) were cultured for 48 hours, followed by cell counting based on absorbance measurement. The fold change relative to mock is shown.
(D) Branched chain amino acid (BCAA) consumption. 2×10^5 cells were cultured for 48 hours and the BCAA concentrations in each medium were measured by colorimetric assays. BCAA consumption was assessed by calculating the difference in BCAA concentrations between the culture medium before and after incubation.
(E) BCAA concentration of the subcutaneous (left) and intrahepatic (right) EMT6 tumors. Tumor cells (1×10^6) were inoculated subcutaneously or intrahepatically on day 0, BCAA concentration was evaluated on day 12. The concentrations were analyzed by colorimetric assays and summary is shown.

All *in vitro* experiments were performed in triplicates and all *in vivo* experiments were performed in duplicate with similar results. One-way analysis of variance with the Bonferroni correction was used in **(A)**, **(B)**, and **(E)**, and unpaired two-tailed Student's *t*-tests were used in **(C)** and **(D)** for statistical analyses. OE: overexpression; ns: not significant; $^{****}P < 0.0001$; bar: mean; error bar: SEM.

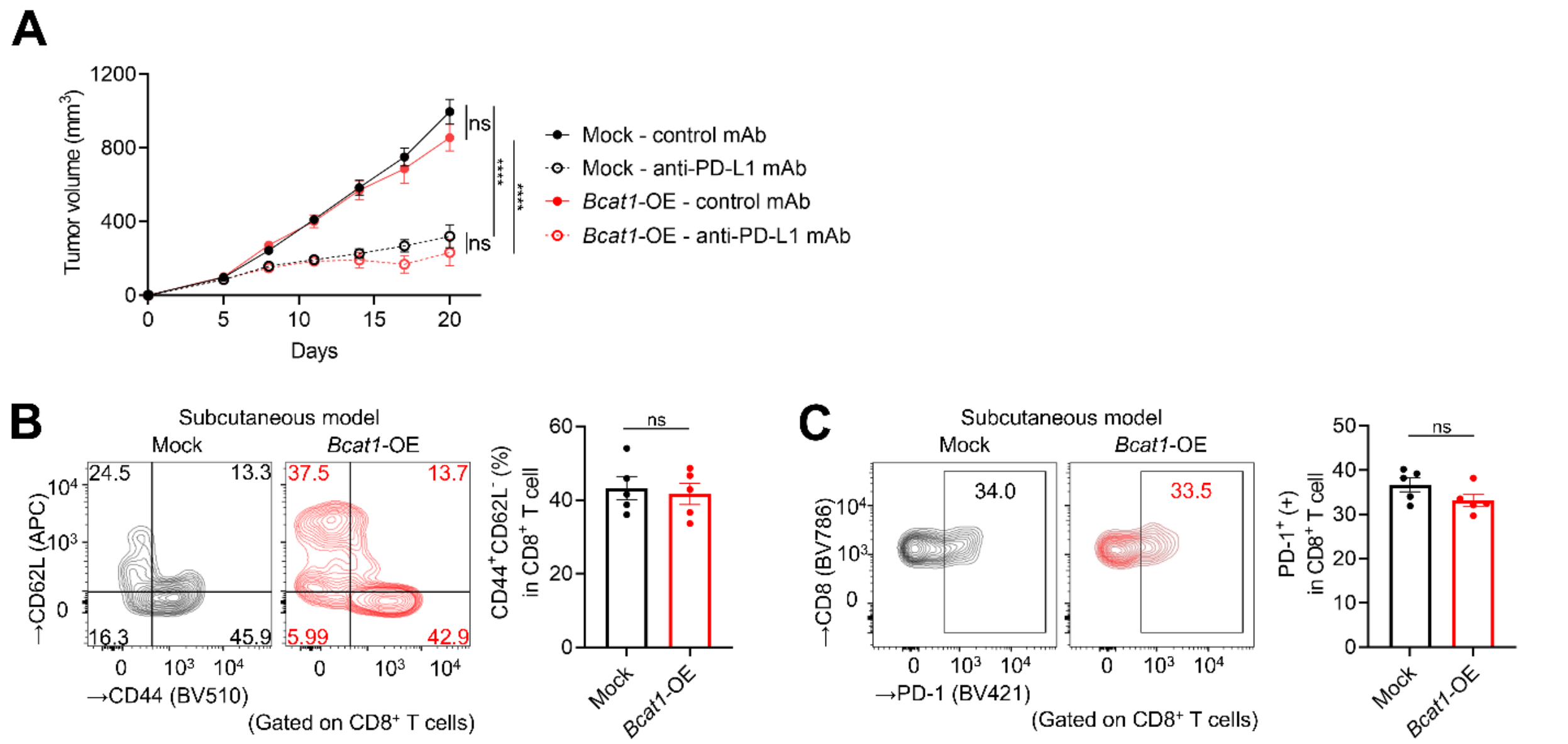

Fig. S9. Additional data related to subcutaneous EMT6 tumor experiments
(A) *In vivo* efficacy of anti-PD-L1 monoclonal antibody (mAb) against subcutaneous EMT6 tumors. Tumor cells (1×10^6) were inoculated subcutaneously on day 0. Each treatment was administered on day 5, 8 and 11 (n = 5 per group) and summary of tumor curves is shown.
(B and **C)** The frequencies of CD44$^+$CD62L$^-$CD8$^+$ effector memory T cells **(B)** and PD-1$^+$CD8$^+$ T cells **(C)** in tumor infiltrating lymphocytes (TILs) of the subcutaneous tumors. Tumors were harvested on day 7 to collect TILs for flow cytometry (FCM) evaluation. Representative FCM staining (left) and summaries (right) are shown.

All *in vivo* experiments were performed in duplicate with similar results. Two-way analysis of variance with the Bonferroni correction was used in **(A)**, and unpaired two-tailed Student's *t*-tests were used in **(B)** and **(C)** for statistical analyses. OE: overexpression; ns: not significant; $^{****}P < 0.0001$; bar: mean; error bar: SEM.

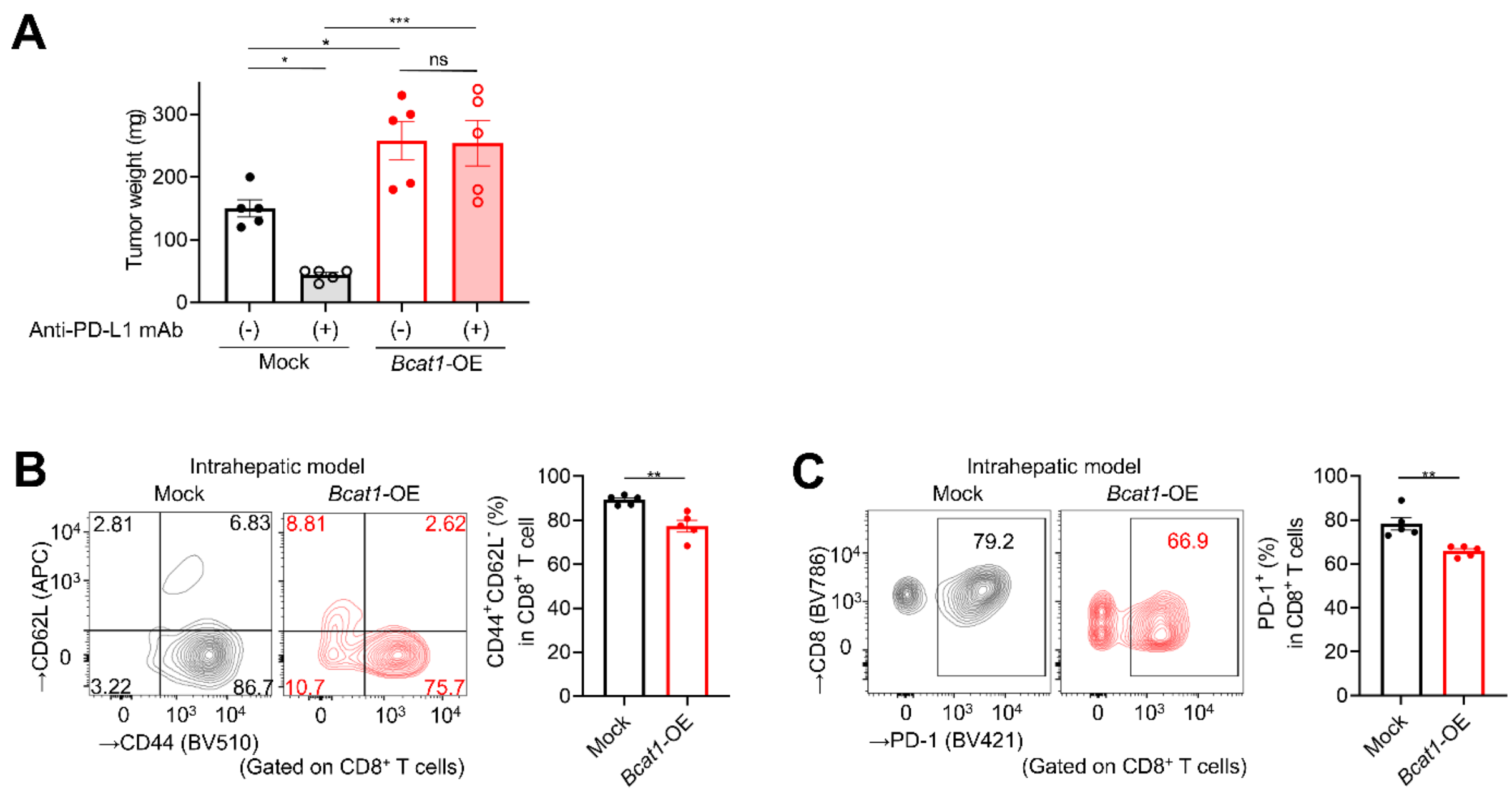

Fig. S10. Additional data related to intrahepatic EMT6 tumor experiments

(A) *In vivo* efficacy of PD-L1 blockade against intrahepatic EMT6 tumors. Tumor cells (1×10^6) were inoculated intrahepatically on day 0. Each treatment was administered on day 5, 8 and 11. Tumor weight was evaluated on day 12 and summary of tumor curves is shown.

(B and C) The frequencies of $CD44^+CD62L^-$ effector memory $CD8^+$ T cells **(B)** and $PD\text{-}1^+CD8^+$ T cells **(C)** in tumor infiltrating lymphocytes (TILs) of the intrahepatic tumors. Tumors were harvested on day 12 to collect TILs for flow cytometry (FCM) evaluation. Representative FCM staining (left) and summaries (right) are shown.

All *in vivo* experiments were performed in duplicate with similar results. Two-way analysis of variance with the Bonferroni correction was used in **(A)**, and unpaired two-tailed Student's *t*-tests were used in **(B)** and **(C)** for statistical analyses. OE: overexpression; ns: not significant; $^{*}P < 0.05$; $^{**}P < 0.01$; $^{***}P < 0.001$; bar: mean; error bar: SEM.

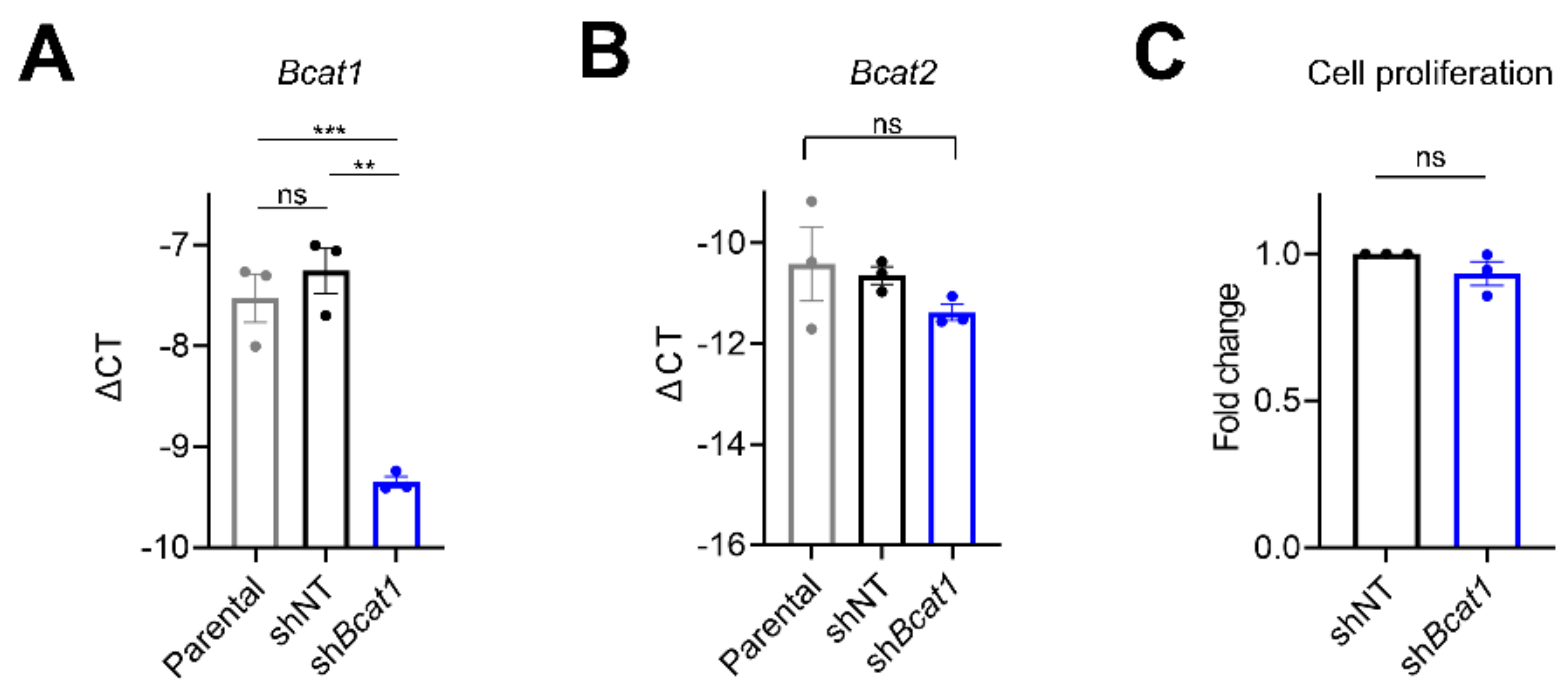

Fig. S11. Additional experimental data related to MC-38 cell lines

(A and **B)** *Bcat1* **(A)** and *Bcat2* **(B)** expression in *Bcat1*-knockdown MC-38 cells using short hairpin RNA. Gene expression was evaluated with reverse transcription quantitative polymerase chain reaction (RT-qPCR). Murine *Actb* was used as an internal control. Each Δcycle threshold (CT) was calculated by subtracting the CT value of each target gene from that of *Actb*, and summaries are shown.

(C) Cell proliferation according to *Bcat1*-knockdown. MC-38 cells (2×10^4 cells) were cultured for 48 hours, followed by cell counting based on absorbance measurement. The fold change relative to non-targeting sort hairpin RNA (shNT) is calculated and summary is shown.

All *in vitro* experiments were performed in triplicates, and all *in vivo* experiments were performed in duplicate with similar results. One-way analysis of variance with the Bonferroni correction was used in **(A)** and **(B)**, and unpaired two-tailed Student's *t*-tests were used in **(C)** for statistical analyses. sh*Bcat1*: *Bcat1* short hairpin RNA; ns: not significant; $^{**}P < 0.01$; $^{***}P < 0.001$; $^{****}P < 0.0001$; bar: mean; error bar: SEM.

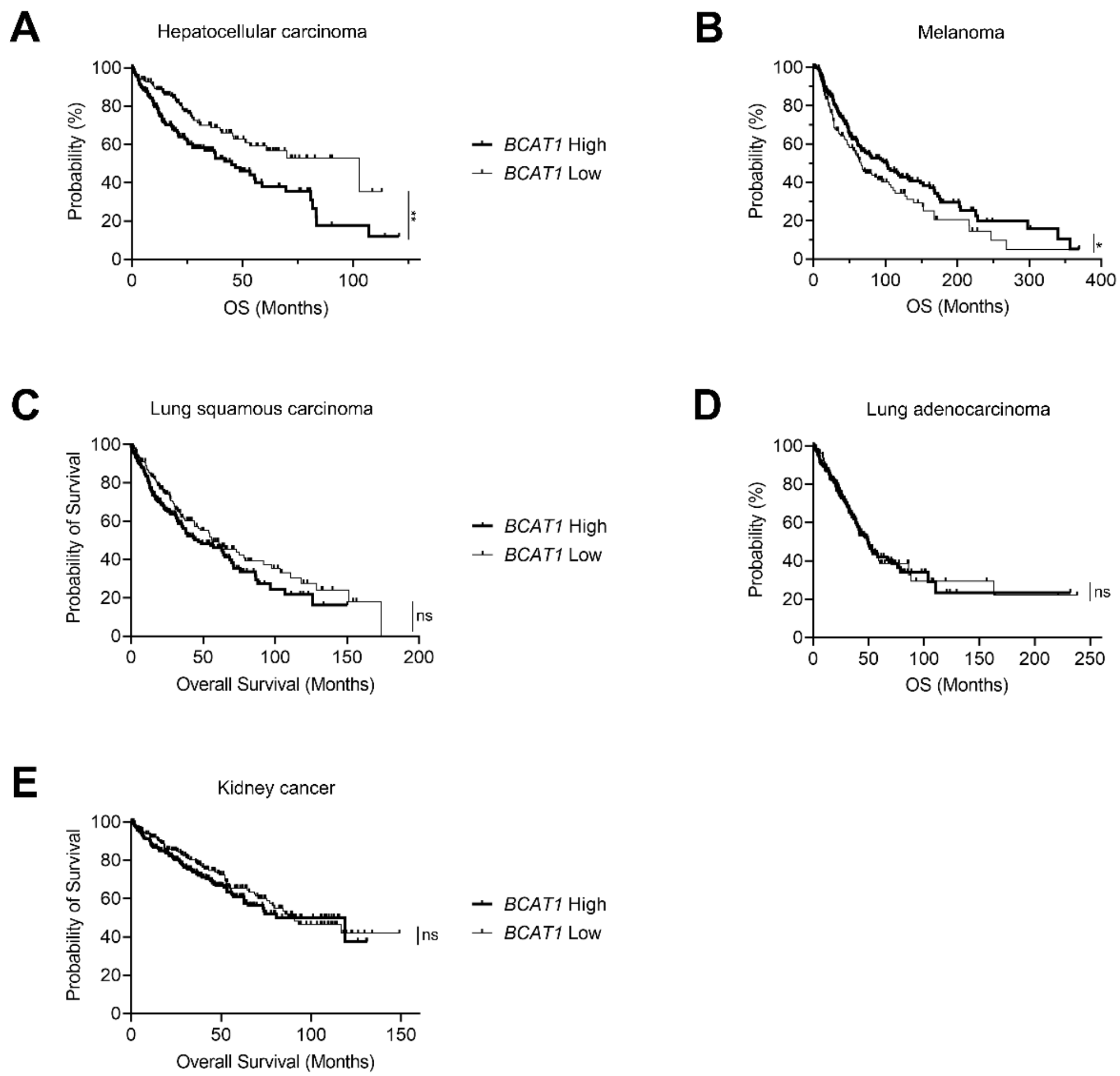

Fig. S12. Prognosis stratified by *BCAT1* expression

(A–E) Kaplan–Meier curves for overall survival (OS) of hepatocellular carcinoma (n = 373) **(A)**, melanoma (n = 462) **(B)**, lung squamous carcinoma (n = 486) **(C)**, lung adenocarcinoma (n = 508) **(D)**, and kidney cancer (n = 496) **(E)**. RNA sequencing data and survival data for each cancer type were acquired from The Cancer Genome Atlas database (Firehose Legacy) published in cBioPortal (https://www.cbioportal.org/). Patients in each cancer type were divided into two groups based on the median of *BCAT1* expression. OS was defined as the time from initiation of therapy to death from any cause.

The log-rank test was used to compare Kaplan–Meier curves for statistical analyses. ns: not significant; $^{*}P < 0.05$; $^{**}P < 0.01$.

Table S1. Baseline characteristics of 50 patients with hepatocellular carcinoma treated with atezolizumab plus bevacizumab

Demographics/characteristics	Number (%)
Sex, male	39 (78)
Age, >72 years	32 (64)
Etiology	
HBV	10 (20)
HCV	15 (30)
ALD	8 (16)
NBNC	25 (50)
MVI, yes	17 (34)
EHM, yes	16 (32)
BCLC stage C	27 (54)
AFP, >400 ng/mL	14 (28)
Performance status	
0	49 (98)
1	1 (2)
Previous systemic treatment	
0	42 (84)
≥1	8 (16)

HBV: hepatitis B virus; HCV: hepatitis C virus; ALD: alcohol-associated liver disease; MASLD: metabolic dysfunction-associated steatotic liver disease; MVI: macrovascular invasion; EHM: extrahepatic metastasis; BCLC: Barcelona clinic liver cancer; AFP: alfa-fetoprotein.

Table S2. Antibodies used for multicolor flow cytometry

Molecule	Clone	Company	Conjugation	RRID
Human CD3	UCHT1	BD Biosciences	BV510	AB_2732053
Human CD4	SK3	BD Biosciences	PE-Cy7	AB_396897
Human CD8	RPA-T8	BD Biosciences	FITC	AB_396580
Human CD45RA	HI100	Biolegend	PerCP-Cy5.5	AB_893357
Human PD-1	MIH4	BD Biosciences	BV421	AB_131322
Human PD-1	EH12.2H7	Biolegend	BV711	AB_11218612
Human CCR7	3D12	BD Biosciences	PE	AB_394354
Human phosphorylated S6K	N7-548	BD Biosciences	Alexa Fluor 488	AB_2869347
Mouse CD3	500A2	BD Biosciences	Alexa Fluor 700	AB_396972
Mouse CD4	RM4-4	Biolegend	PerCP-Cy5.5	AB_2563022
Mouse CD8	53-6.7	BD Biosciences	FITC	AB_394568
Mouse CD8	53-6.7	Biolegend	BV785	AB_11218801
Mouse CD62L	MEL-14	Biolegend	APC	AB_313098
Mouse CD62L	MEL-14	Biolegend	BV711	AB_2564215
Mouse CD44	IM7	BD Biosciences	BV510	AB_2738011
Mouse PD-1	J43	BD Biosciences	BV421	AB_2737668
Mouse SLAMF6	330-AJ	BD Biosciences	PE	AB_2188095
Mouse TIM3	B8.2C12	Biolegend	PE-Cy7	AB_2632734
Mouse Granzyme B	QA16A02	Biolegend	FITC	AB_2687030
Mouse INF-γ	XMG1.2	Biolegend	BV421	AB_2738165
Viability dye	-	ThermoFisher	eFluor 780	-

Table S3. Primer list

**Name**	**Sequence**
Bcat1 - Forward	CTGCCTCTGTTTTGCACTACGC
Bcat1 - Reverse	TCCTCACAGCAGATCGGCACAT
Bcat2 - Forward	CAAAGGTGGAGACCAGCAGGTA
Bcat2 - Reverse	TGGCGGATACACTCCAACAGCT
Actb - Forward	CATTGCTGACAGGATGCAGAAGG
Actb - Reverse	TGCTGGAAGGTGGACAGTGAGG

Table S4. KEGG pathways significantly upregulated in tumors with high PD-1–expressing CD8^{+} tumor-infiltrating T cells (*P* value <0.05 and false discovery rate [FDR] <0.05)

No.	Name	NES	*P* value	FDR
1	Ribosome	4.34	0.00	0.00
2	Valine, leucine and isoleucine degradation	3.79	0.00	0.00
3	Citrate cycle (TCA cycle)	3.75	0.00	0.00
4	Fatty acid metabolism	3.71	0.00	0.00
5	Protein export	3.70	0.00	0.00
6	Parkinson’s disease	3.68	0.00	0.00
7	Ascorbate and aldarate metabolism	3.66	0.00	0.00
8	Peroxisome	3.56	0.00	0.00
9	Propanoate metabolism	3.34	0.00	0.00
10	Proteasome	3.27	0.00	0.00
11	Complement and coagulation cascades	3.22	0.00	0.00
12	Retinol metabolism	3.21	0.00	0.00
13	Drug metabolism – cytochrome P450	3.13	0.00	0.00
14	Porphyrin and chlorophyll metabolism	2.95	0.00	0.00
15	Pentose and glucuronate interconversions	2.93	0.00	0.00
16	N-glycan biosynthesis	2.92	0.00	0.00
17	Glycine serine and threonine metabolism	2.91	0.00	0.00
18	Steroid hormone biosynthesis	2.79	0.00	0.00
19	Butanoate metabolism	2.79	0.00	0.00
20	Oxidative phosphorylation	2.73	0.00	0.00
21	Drug metabolism other enzymes	2.72	0.00	0.00
22	Beta alanine metabolism	2.64	0.00	0.00
23	Arginine and proline metabolism	2.54	0.00	0.00
24	Tryptophan metabolism	2.48	0.00	0.00
25	Metabolism of xenobiotics by cytochrome P450	2.48	0.00	0.00
26	Alzheimer’s disease	2.47	0.00	0.00
27	Lysine degradation	2.31	0.00	0.00
28	Biosynthesis of unsaturated fatty acids	2.27	0.00	0.00
29	Primary bile acid biosynthesis	2.25	0.00	0.00
30	Pyruvate metabolism	2.23	0.00	0.00
31	Glyoxylate and dicarboxylate metabolism	2.16	0.01	0.00
32	Nucleotide excision repair	2.15	0.00	0.00
33	Alanine aspartate and glutamate metabolism	1.99	0.00	0.00
34	Histidine metabolism	1.97	0.00	0.00
35	Starch and sucrose metabolism	1.90	0.00	0.00
36	Mismatch repair	1.89	0.00	0.00
37	PPAR signaling pathway	1.88	0.00	0.00
38	Glutathione metabolism	1.80	0.00	0.01
39	Glycosylphosphatidylinositol GPI anchor biosynthesis	1.76	0.00	0.01
40	Phenylalanine metabolism	1.76	0.00	0.01
41	Thyroid cancer	1.64	0.00	0.02
42	Terpenoid backbone biosynthesis	1.57	0.02	0.03

43	Glycolysis gluconeogenesis	1.50	0.00	0.04
44	Tyrosine metabolism	1.49	0.03	0.04
45	Cysteine and methionine metabolism	1.49	0.02	0.04

NES: normalized enrichment score.

Table S5. KEGG pathways significantly downregulated in tumors with high PD-1–expressing CD8$^+$ tumor-infiltrating T cells (*P* value <0.05 and false discovery rate [FDR] <0.05)

No.	Name	NES	*P* value	FDR
1	Olfactory transduction	-2.00	0.00	0.00
2	Dilated cardiomyopathy	-1.71	0.00	0.02
3	Taste transduction	-1.64	0.00	0.04

NES: normalized enrichment score.

Table S6. KEGG pathways significantly upregulated in tumors from responders treated with atezolizumab and bevacizumab (*P* value <0.05 and false discovery rate [FDR] <0.05)

No.	Name	NES	*P* value	FDR
1	Ribosome	5.46	0.00	0.00
2	Oxidase phosphorylation	3.84	0.00	0.00
3	Parkinson’s disease	3.83	0.00	0.00
4	Ascorbate and aldarate metabolism	3.46	0.00	0.00
5	Drug metabolism – other enzymes	3.31	0.00	0.00
6	Pentose and glucuronate interconversions	3.18	0.00	0.00
7	Complement and coagulation cascades	3.08	0.00	0.00
8	Peroxisome	3.06	0.00	0.00
9	Retinol metabolism	3.05	0.00	0.00
10	Citrate cycle (TCA cycle)	3.03	0.00	0.00
11	Protein export	3.01	0.00	0.00
12	Terpenoid backbone biosynthesis	2.98	0.00	0.00
13	Valine, leucine and isoleucine degradation	2.86	0.00	0.00
14	Alzheimer’s disease	2.82	0.00	0.00
15	Steroid hormone biosynthesis	2.80	0.00	0.00
16	Drug metabolism cytochrome P450	2.79	0.00	0.00
17	Propanoate metabolism	2.65	0.00	0.00
18	Porphyrin and chlorophyll metabolism	2.60	0.00	0.00
19	Metabolism of xenobiotics by cytochrome P450	2.51	0.00	0.00
20	Fatty acid metabolism	2.45	0.00	0.00
21	Primary bile acid biosynthesis	2.31	0.00	0.00
22	Proteasome	2.30	0.00	0.00
23	N-glycan biosynthesis	2.20	0.00	0.00
24	Steroid biosynthesis	2.20	0.01	0.00
25	Starch and sucrose metabolism	2.09	0.00	0.00
26	Glutathione metabolism	2.09	0.00	0.00
27	Biosynthesis of unsaturated fatty acids	1.93	0.00	0.01
28	Pyruvate metabolism	1.77	0.00	0.01
29	Butanoate metabolism	1.71	0.00	0.02
30	Maturity onset diabetes of the young	1.60	0.04	0.03
31	Pantothenate and COA biosynthesis	1.56	0.04	0.04
32	Amino sugar and nucleotide sugar metabolism	1.55	0.00	0.04

NES: normalized enrichment score.

Table S7. KEGG pathways significantly downregulated in tumors from responders treated with atezolizumab and bevacizumab (*P* value <0.05 and false discovery rate [FDR] <0.05)

No.	Name	NES	*P* value	FDR
1	Olfactory transduction	-2.14	0.00	0.00
2	Alpha-linolenic acid metabolism	-1.82	0.00	0.02

NES: normalized enrichment score.
